# A first comprehensive analysis of Transcribed Ultra Conserved Regions uncovers important regulatory functions of novel non-coding transcripts in gliomas

**DOI:** 10.21203/rs.3.rs-4164642/v1

**Published:** 2024-04-18

**Authors:** Myron K Gibert, Ying Zhang, Shekhar Saha, Pawel Marcinkiewicz, Collin Dube, Kadie Hudson, Yunan Sun, Sylwia Bednarek, Bilhan Chagari, Aditya Sarkar, Christian Roig-Laboy, Natalie Neace, Karim Saoud, Initha Setiady, Farina Hanif, David Schiff, Pankaj Kumar, Benjamin Kefas, Markus Hafner, Roger Abounader

**Affiliations:** 1 University of Virginia Department of Microbiology, Immunology & Cancer Biology, Charlottesville, VA, 22908, USA; 2 University of Virginia Department of Neurology, Charlottesville, VA, 22908, USA; 3 University of Virginia Department of Cancer Center, Charlottesville, VA, 22908, USA; 4 University of Virginia Department of Public Health Sciences and Bioinformatics Core, Charlottesville, VA, 22908, USA; 5 National Institutes of Health, Bethesda, MD, USA

## Abstract

Transcribed Ultra-Conserved Regions (TUCRs) represent a severely understudied class of putative non-coding RNAs (ncRNAs) that are 100% conserved across multiple species. We performed the first-ever analysis of TUCRs in glioblastoma (GBM) and low-grade gliomas (LGG). We leveraged large human datasets to identify the genomic locations, chromatin accessibility, transcription, differential expression, correlation with survival, and predicted functions of all 481 TUCRs, and identified TUCRs that are relevant to glioma biology. Of these, we investigated the expression, function, and mechanism of action of the most highly upregulated intergenic TUCR, uc.110, identifying it as a new oncogene. Uc.110 was highly overexpressed in GBM and LGG, where it promoted malignancy and tumor growth. Uc.110 activated the WNT pathway by upregulating the expression of membrane frizzled-related protein (MFRP), by sponging the tumor suppressor microRNA miR-544. This pioneering study shows important roles for TUCRs in gliomas and provides an extensive database and novel methods for future TUCR research.

## INTRODUCTION

Transcribed Ultra-conserved Regions (TUCRs) represent 481 unique transcribed RNA molecules that are “ultraconserved” across multiple species, including in the human, mouse (100%), rat (100%), dog (98%), and chicken (95%) genomes. [1] TUCR expression has been found to be highly deregulated in some cancers. Because of their ultra-conservation and their deregulation, it is believed that TUCRs may have important regulatory roles in cancer. [2–11] About 90% of the genome is transcribed, but only ~2 percent of the transcriptome is translated. The remainder of the transcriptome is made up of non-coding elements that serve key regulatory roles. Of these elements, long non-coding RNAs (lncRNAs) serve as important regulators of malignancy and potential therapeutic targets in cancer. [2, 12–19] Due to their size and lack of known associated protein products, it has been suggested that many TUCRs may function as lncRNAs.[2] The putative existence of “ultraconserved” lncRNAs is significant, as lncRNAs are typically poorly conserved as a class of molecules.[2] Very little is known about TUCRs. [2] In particular, the literature elucidating the expressions, functions, and mechanisms of action of TUCRs in glioblastoma (GBM) and low-grade glioma (LGG) is nonexistent. GBM and LGG represent over 80% of primary malignant brain tumors in humans, of which GBM is the deadliest, with a median survival of approximately 15 months. [20–28] Studying TUCRs in gliomas is therefore an untouched avenue for understanding novel oncogenic mechanisms and discovering new biomarkers and therapeutic targets.

**Figure 1.**
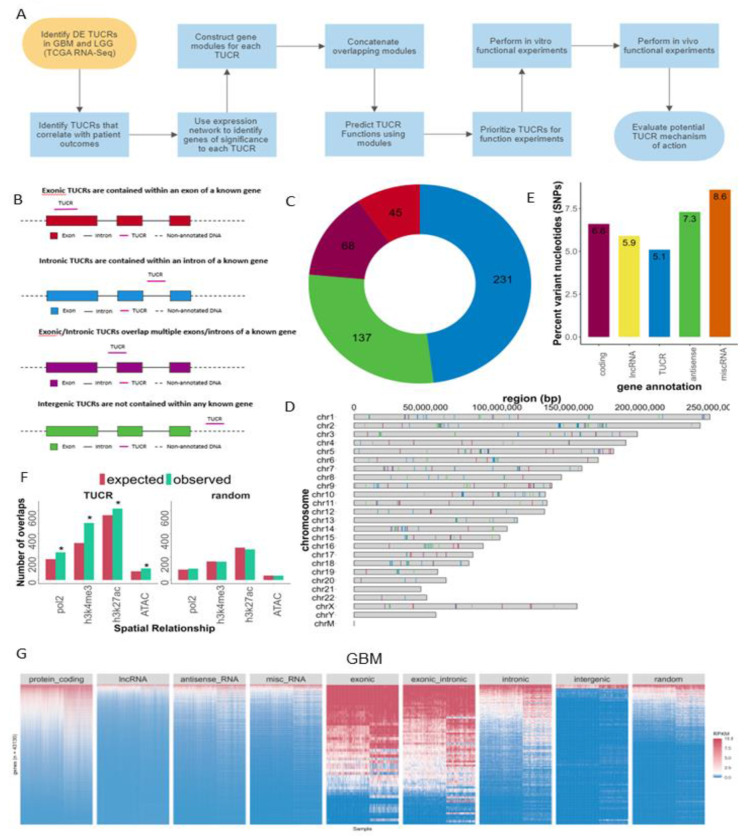
Annotation, localization, and expression of TUCRs in GBM and LGG. A) Experimental workflow for identifying and studying TUCRs of interest. B) TCGA analysis shows that TUCRs can be exonic (red), intronic (blue), exonic/intronic (purple) or intergenic (green). C) Circle graph showing the distribution of genomic annotations across all 481 TUCRs, with colors matching 1B. D) Karyoplot showing that TUCRs exist on all chromosomes except for Chr21, the Y chromosome and mitochondrial DNA, vertical lines show TUCRs with colors matching 1B. E) Bar chart demonstrating that TUCRs are more resistant to single nucleotide variants (SNVs/SNPs) than other gene annotation categories. F) Bar chart showing that TUCRs are enriched for markers for open and active chromatin in GBM cells, suggesting that they represent transcriptionally active sites. Red bars represent chi-square expected overlaps, and teal bars represent observed values. G) Heatmap representing TUCR absolute expression (RPKM) across multiple gene annotations. Blue represents poorly expressed genes (<1 RPKM), White/Pink genes are moderately expressed (>=1 RPKM) and Red represents highly expressed genes (RPKM >=10). TUCRs demonstrate an expression profile that is most comparable with protein coding genes. * = p < 0.05

In this study, we leveraged large human datasets to identify the genomic locations, chromatin accessibility, transcription, differential expression, correlation with survival, and predicted functions of all 481 TUCRs, and identified TUCRs that are relevant to glioma biology (Figure 1A). Of these, we investigated the expression, function, and mechanism of action of the most highly upregulated intergenic TUCR, uc.110, identifying it as a new oncogene. Uc.110 was highly overexpressed in GBM and LGG, where it promoted malignancy parameters and tumor growth. Uc.110 activated the WNT pathway by upregulating the expression of membrane frizzled-related protein (MFRP), by sponging the tumor suppressor microRNA miR-544. This work shows important roles for TUCRs in gliomas and provides an extensive database and novel methods for future TUCR research in any disease context.

## RESULTS

### TUCRs are encoded throughout the genome, resistant to variation, and actively transcribed.

We analyzed TUCR genomic locations published in Bejerano et al. [1] using hg38 genome coordinates lifted over from the provided hg19 coordinates. We found that some TUCRs are exonic and are contained within an exon of the “host” gene. Others are contained within an intron. Some TUCRs straddle a region that spans exonic and intronic regions of the host gene (exonic/intronic), and others are not contained within any known genetic element (intergenic) (Figure 1B). We manually annotated each TUCR using a combination of UCSC Genome Browser tracks, [29, 30] Quinlan Laboratory’s bedtools, [31, 32] and TUCR genomic locations lifted over to hg38 from hg19. [1] We identified 45 exonic, 231 intronic, 68 intronic/exonic, and 137 intergenic TUCRs (Figure 1C). We found that TUCRs are located on all but one 21 numbered chromosomes and the X chromosome. There were no annotated TUCRs on chromosome 21 (chr21), the Y chromosome (chrY) or in the mitochondrial DNA (chrM) (Figure 1D). Detailed TUCR annotation information for every single TUCR is provided in the supplementary materials (Supplementary Master Table).

Since TUCRs are expected to be resistant to variation [2], we characterized the overlap of current dbSNP (build 156) single nucleotide polymorphism (SNP) annotations to the lifted over hg38 TUCR genomic coordinates. We found that TUCRs overlap with fewer SNPs than protein coding genes and non-coding RNAs, indicating that they are more resistant to variation (Figure 1E).

We also investigated TUCR transcription levels in comparison to transcription of known protein-coding and non-coding genes. To accomplish this, we first analyzed their spatial associations with markers for active chromatin (H3K4me3), active enhancers (H3K27ac), lncRNA transcription (RNA Pol.II) and open chromatin (ATAC-Seq). We determined the significance of the spatial relationships between these marks and TUCRs utilizing publicly available U87 CHIP- and ATAC-Seq datasets. Then, we compared the data to TUCR intervals that were randomly shuffled to create a negative control, other classes of non-coding RNAs, and TUCRs subset by genomic annotation (Figure 1F and Supplementary Figure 1A). We found that TUCRs displayed a significant enrichment for all transcriptional activity markers over expected and compared to control. The above data show that TUCRs are distributed throughout the genome, resistant to variation, and actively transcribed in GBM and LGG.

### TUCRs are highly expressed in GBM and LGG tumors.

TUCR expression has not been characterized in GBM or LGG before. We performed the first comprehensive analysis of TUCR expression in these cancers by comparing GBM (n = 166) and LGG (n = 505) tumor samples from the Cancer Genome Atlas (TCGA) [33] to their normal brain cortex counterparts in TCGA (n = 5) and the Genotype-Tissue Expression Database (GTEx, n = 255). [34] We first analyzed absolute TUCR expression, as measured by reads-per-kilobase million (RPKM). The absolute expression, in GBM, of all TUCRs was compared to the expression of lncRNAs, coding genes, antisense RNAs, and small noncoding RNAs (< 200 nt length), and the expression of TUCRs separated by genomic annotation into exonic, intronic, exonic/intronic, and intergenic. All gene annotations were obtained using the CHESS gene catalog, which contains most Refseq and Ensembl genes, while also including a series of understudied novel genes.[35] Highly expressed genes are visualized via heatmap (>=10 RPKM, red) along with moderately (>=1 RPKM, white) and lowly expressed genes (<1 RPKM, blue). These analyses were repeated in LGG (Supplementary Figure 1B). The data show that intragenic TUCRs are expressed at magnitudes that are like those of protein coding genes in both GBM and LGG, while intergenic TUCRs demonstrate expression levels that are closer to those of lncRNAs (Figure 1G).

### TUCRs are deregulated in gliomas, and deregulation is associated with clinical outcomes.

**Figure 2.**
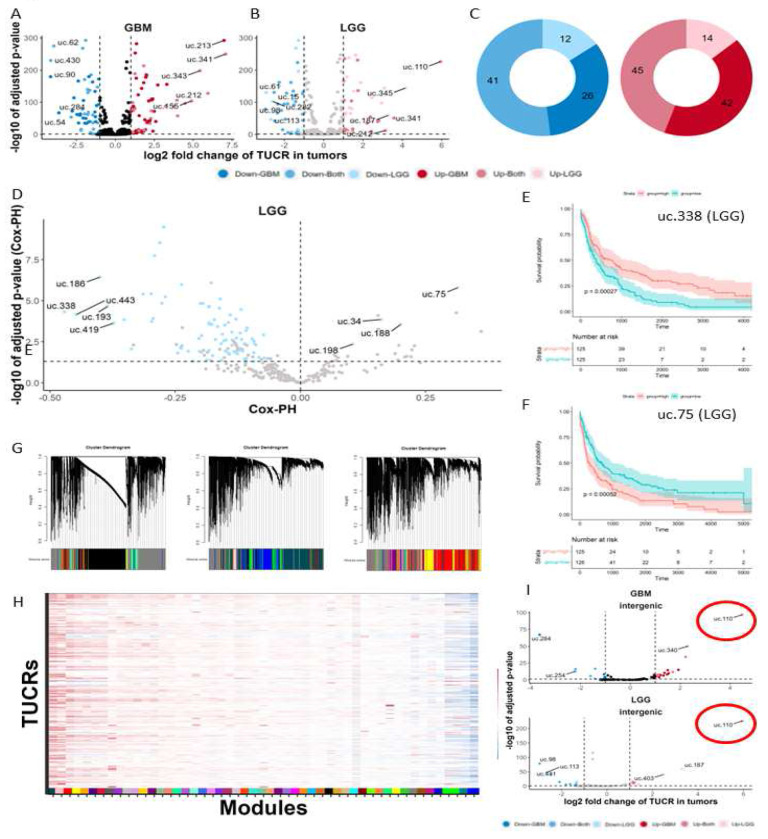
TUCRs are deregulated and associated with patient outcomes in GBM and LGG and may have broad functional roles. All experiments were performed using TCGA GBM and LGG and GTEx normal brain RNA-Seq data. A) Volcano plot showing that 87 TUCRs are upregulated >=2-fold (1-log2FC) and 67 are downregulated in GBM. Red dots are upregulated. Blue dots are downregulated. B) Volcano plot showing that 59 TUCRs are upregulated >=2-fold in LGG, and 53 are downregulated in LGG. C) Circle graph demonstrating that of the 154 deregulated TUCRs in GBM, 86 were also deregulated in LGG, a 56% overlap. Dark Red/Blue are TUCRs deregulated in GBM. Light Pink/Blue are TUCRs deregulated in LGG. Intermediate Red/Blue represent TUCRs deregulated in both. D) Volcano plot showing that several TUCRs are significantly associated with patient outcomes in LGG. Pink dots represent TUCRs significantly associated with poor prognosis. Blue dots represent TUCRs significantly associated with good prognosis. E) Kaplan-Meier showing that TUCR uc.338 is significantly associated with good prognosis. Red line represents the uc.338 high expression group. Teal line represents the uc.338 low expression group. F) Kaplan-Meier showing that uc.75 is significantly associated with poor prognosis (Line colors as described in E). G) Gene similarity dendrograms from weighted gene correlation network analysis (WGCNA). 42,644 genes were aggregated into 3 "blocks" by gene similarity and were then further aggregated into 51 linkage modules using TUCR expression as trait data. Modules are denoted with distinct color hex codes. (e.g. #004C54 is the “midnight green” module). H) Heatmap showing that TUCRs demonstrate association with all 60 modules, suggesting broad potential functions. Red and Blue represent positive and negative correlations, respectively. I) Volcano plot showing that the uc.110 TUCR is the most upregulated TUCR in GBM and LGG (Line colors as described in E). * = p < 0.05

We analyzed TCGA tumor data and GTEx normal brain cortex data and found that in addition to being highly expressed in gliomas, TUCRs are highly deregulated in GBM and LGG as compared to normal brain cortex. Of the 481 annotated TUCRs, we identified 87 that were upregulated and 67 that were downregulated in GBM (Figure 2A). We also identified 59 TUCRs that were upregulated and 53 TUCRs that were downregulated in LGG. (Figure 2B). Of the 154 deregulated TUCRs in GBM, 86 were also deregulated in LGG, a 56% overlap (Figure 2C). We then sought to determine whether deregulation of TUCR expression correlates with patient outcomes in GBM and LGG. For each of the 481 TUCRs, we generated a Kaplan-Meier plot tracking differences in survival for high expressing (upper quartile) and low expressing (lower quartile) tumor groups. Of the TUCRs that are expressed in GBM TCGA RNA-Seq data, only 4 were correlated with survival in a statistically significant manner in both of our workflows (p <= 0.05, Supplementary Figure 2B). We considered that this low prevalence of survival associated TUCRs in GBM was due to the short overall survival of GBM patients (~15 months). We also studied survival differences in LGG patients, as they have a longer median survival (~84 months). Of the TUCRs that are expressed in LGG TCGA RNA-Seq data, 93 were correlated with survival in both of our workflows (Figure 2D). We have highlighted two TUCRs that represent a statistically significant correlation with good (uc.338, Figure 2E) or poor (uc.75, Figure 2F) prognosis using both methods. When separated by annotation category, intragenic TUCR deregulation has a greater association with patient outcomes than intergenic TUCR deregulation (Supplementary Figure 2C). Expression, deregulation, and survival analyses were performed on all 481 TUCRs. Detailed results for individual TUCRs can be found at http://www.abounaderlab.org/tucr-database/.

### TUCRs are coregulated with genes that have specific functions.

We predicted TUCR functions by identifying coregulated genes with known functions via weighted gene co-expression network analysis (WGCNA).[36] We aggregated the 42,644 genes in our dataset into 60 colored modules based on clustered gene ontology (GO) terms. Each of these modules contains genes with known functions, such as RNA binding, cell signaling, immune response, metabolic response, etc. These modules can also be used to identify genes that associate with clinical traits, such as the tumor tissue-type (Figure 2G). The data can also be used to predict gene function for novel genes. To do this, we aggregated all 481 TUCRs into our modules. We identified TUCRs that correlate with each of the 60 modules, with some having positive correlations and others negative. For example, TUCRs that exhibit a positive correlation with the #004C54 “midnight green” module (Supplementary Figure 6) could have a promoting effect on nucleic acid binding and regulation, while those that are negatively correlated with the #f4a460 module (Supplementary Figure 7) may have a negative effect on G-protein coupled receptor and metabolic functions. Since many different TUCRs show associations with different modules, and every module has at least one TUCR that is associated with it, these results suggest that TUCRs may have a broad range of potential functions in GBM and LGG (Figure 2H). WGCNA analyses were performed on all 481 TUCRs. Detailed results for individual TUCRs can be found at http://www.abounaderlab.org/tucr-database/.

### TUCR, uc.110, is highly upregulated in gliomas and is predicted to bind nucleic acids.

**Figure 3.**
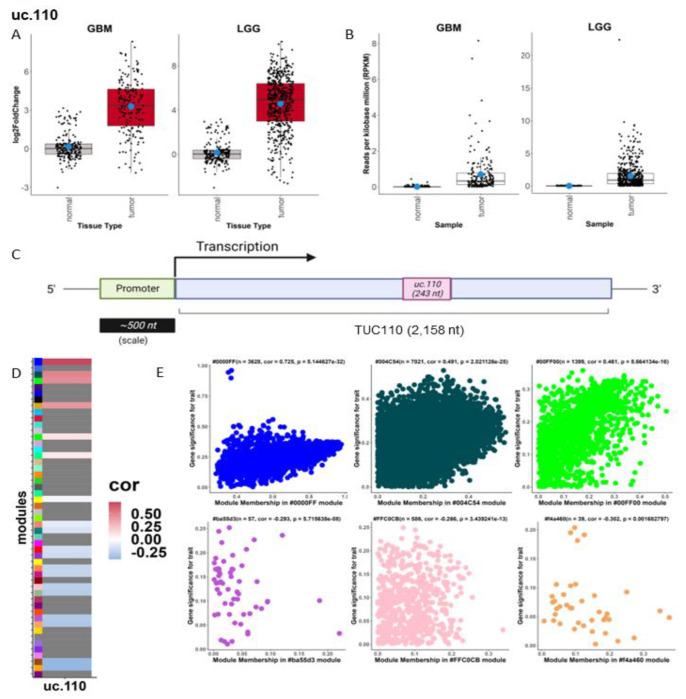
The uc.110 TUCR is the most upregulated intergenic TUCR in gliomas and is predicted to bind nucleic acids. A) Box- and dotplot showing that uc.110 is 30-fold upregulated in GBM and ~60-fold upregulated in LGG based on TCGA and GTEx data analyses. Red boxes represent upregulated TUCRs. B) Box- and dotplot showing that uc.110 is expressed in tumors but is poorly expressed in normal brain cortex based on TCGA and GTEx data analyses. C) Cartoon showing that uc.110 is a 243 nt region in a 2,158 nt transcript. D) Heatmap depicting uc.110 gene module association. Positive correlations are red, while negative correlations are blue, with weak correlations in white. Modules with no linkage are gray. E) Scatter plots depicting uc.110 association with top 3 positive (top row) and negative (bottom row) correlation modules. Caption lists the module name, number of genes in the module, and the significance of uc.110 association with the module.

The expression and deregulation of intergenic TUCRs is of particular interest as they may represent novel lncRNAs due to their similar expression levels, genomic location, and lack of coding potential.[2] These TUCRs are also easier to study experimentally; they are often thousands of kilobases (kb) from the nearest protein-coding gene and likely function in a manner that is independent of a “host gene”. Because of this, we focused on intergenic TUCRs for our experimental studies. Of the deregulated intergenic TUCRs in GBM and in LGG (Figure 2I), we found that uc.110 is the most upregulated as compared to normal brain; 30-fold in GBM and 61.4-fold in LGG (Figure 3A). It has near binary expression; it is rarely expressed at all in normal brain but is very highly expressed in GBM and LGG (Figure 3B). Due to its high expression, we hypothesized that this TUCR is functioning as an oncogene.

Since many TUCRs exist as a part of a larger transcript [2], we first determined the sequence of the uc.110 full transcript. We utilized machine learning and *de novo* transcript reassembly using TCGA and GTEx RNA-seq data to reconstruct RNA-Seq transcripts in the absence of a reference genome (Supplementary Figure 3A). [35] We identified a 2,158 nucleotide (nt) long RNA molecule that contains the 243 nucleotide (nt) uc.110 ultraconserved sequence (Figure 3C) as a novel transcript. We confirmed the existence of this transcript experimentally using PCR amplifications and sequencing (Supplementary Figure 3B).

After identifying the sequence for the full uc.110 transcript (Supplementary Figure 3C), we utilized our WGCNA workflow to identify genes and modules (Figure 3D) that are significant to this transcript. Of note, one of the top modules for uc.110 by module association is the #004C54 module, which represents genes that are involved in nucleic acid and protein binding (Supplementary Figure 6). This is a published function for some TUCRs. [2] Genes that are members of these modules are positively coregulated with uc.110 (Figure 3E). Based on these findings, we hypothesized that uc.110 may be operating as an oncogenic RNA-binding molecule. We also performed similar analyses for all 481 TUCRs to identify potential functional roles for each TUCR in gliomas. Examples of an oncogenic TUCR (uc.2, Supplementary Figure 5) and a tumor suppressor TUCR (uc.15, Supplementary Figure 5) are depicted in this manuscript, while the analyses of the rest of the 481 TUCRs can be found at http://www.abounaderlab.org/tucr-database/.

### uc.110 has oncogenic effects in GBM.

**Figure 4.**
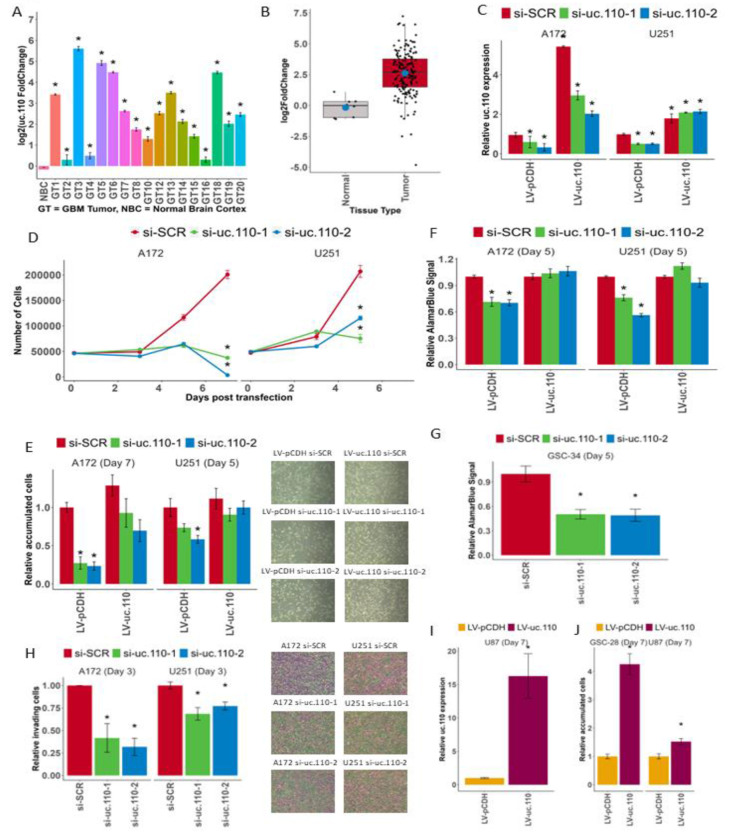
The uc.110 TUCR operates as an oncogene. A) Bar graph depicting uc.110 upregulation in banked UVA GBM tumors versus normal brain cortex. B) Boxplot representing uc.110 expression in pooled tumors versus normal brain. Red boxes indicate an upregulated TUCR. C) Bar graph depicting qPCR validation of uc.110 siRNA knockdown and rescue in A172 and U251 cell lines. Facets represent cell lines. si-SCR = scrambled control siRNA (red), si-uc. 110-1 = siRNA targeting uc. 110 at nucleotide 96/243 (green), si-uc.110-2 = siRNA targeting uc.110 at nucleotide 195/243 (blue). D) Line graph showing that knockdown of uc.110 reduces A172 and U251 cell accumulation over a 5–7 day period. Facets represent cell lines. E) Bar graph depicting that the cell accumulation phenotype is rescued when uc.110 is overexpressed in the presence of siRNA. Facets represent cell lines. Images are representative of the listed sample. F) Bar graph showing that knockdown of uc.110 reduces A172 and U251 cell viability via Alamar Blue assay and can be rescued with uc.110 overexpression. Facets represent cell lines. G) Bar graph showing that knockdown of uc.110 reduces GSC-34 glioma stem cell viability via Alamar Blue. H) Bar graph showing knockdown of uc.110 reduces A172 and U251 cell invasion and migration. Images are representative of the listed sample. I) Bar graph showing the qPCR validation of overexpression in U87 cells. J) Bar graph showing that overexpression of uc.110 increases cell accumulation in U87 and GSC-28 cells. Facets represent cell lines. * = p < 0.05

To determine the function of uc.110 in GBM, we first used qPCR to investigate the expression of uc.110 in our banked tumor samples compared to normal brain cortex and cell lines compared to normal human astrocytes. We independently confirmed the results from our TCGA analysis by showing uc.110 is highly upregulated in GBM tumors (Figure 4A, 4B and Supplementary Figure 8). We then designed two siRNAs that target separate regions on the uc.110 RNA, one that begins at nucleotide 96/243 (si-uc. 110-1) and one that begins at nucleotide 195/243 (si-uc.110-2), as well as a scrambled control (si-SCR) (Supplementary Figure 8A). We generated stable A172 and U251 GBM cell lines that express uc.110 (LV-uc.110) or the empty expression vector (LV-pCDH). We subjected these cell lines to siRNA transfection and assessed the effects on cell counting, survival and invasion assays (Supplementary Figure 8B). We used qPCR to show that uc.110 is generally, though not uniformly, upregulated in GBM cells (Supplementary Figure 8). Based on these data, we prioritized the use of cell lines that overexpress uc.110 (A172, U251) for knockdown experiments, and cells that express low levels of uc.110 (U87, GSC-28) for overexpression experiments. We confirmed that siRNAs targeting of uc.110 lead to knockdown of uc.110 expression in A172 and U251 cells. (Figure 4C) We also confirmed that LV-uc.110 overexpresses uc.110, and that this overexpression rescues uc.110 bioavailability in A172 and U251 cells (Figure 4C).

Next, we performed cell counting assays [20, 37–39] to determine the effects of uc.110 knockdown and rescue on cell accumulation. When we reduced uc.110 expression, we reduced cell accumulation in A172 and U251 cells (Figure 4D). When we rescued uc.110 bioavailability by restoring its expression, the cell accumulation phenotype was restored in A172 and U251 cells (Figure 4E). We then used AlamarBlue [40, 41] to measure cell viability. When we reduced uc.110 expression, A172 and U251 cell viability was reduced. We were able to rescue this phenotype by increasing uc.110 bioavailability (Figure 4F). We observed a similar phenotype in a glioma stem cell line that overexpresses uc.110 (GSC-34, Figure 4G).

We then investigated the invasive potential of uc.110 using a transwell invasion assay. [42–44] Knockdown of uc.110 reduced A172 and U251 cell invasion through a collagen IV matrix (Figure 4H). When uc.110 bioavailability was increased, a partial recovery of the phenotype was observed (Supplementary Figure 8F). Lastly, we overexpressed uc.110 in U87 and GSC-28 cells (Figure 4I) and determined that this leads to increased cell accumulation compared to the empty vector after 7 days (Figure 4J) in U87 and GSC-28 cells. These data show that uc.110 has oncogenic effects in GBM cells and stem cells.

**Figure 5.**
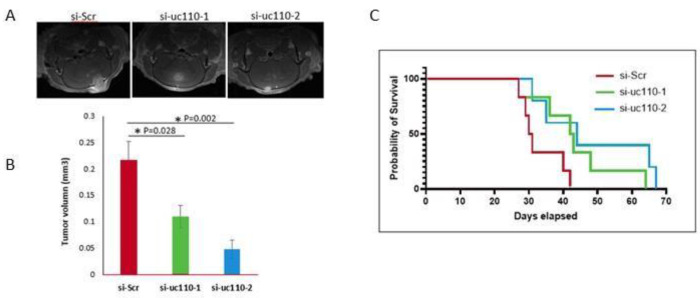
The uc.110 TUCR promotes tumor growth in vivo. A) MRI images reveal a reduction in tumor size when uc.110 is knocked down via siRNAs. B) Bar graph showing that knockdown of uc.110 leads to a reduction in tumor volume. si-SCR = scrambled control siRNA (red), si-uc.110-1 = siRNA targeting uc. 110 at nucleotide 96/243 (green), si-uc.110-2 = siRNA targeting uc.110 at nucleotide 195/243 (blue). C) Kaplan-Meier plot showing that knockdown of uc.110 leads to increased mouse survival.

After determining that uc.110 displays an oncogenic phenotype *in vitro*, we sought to determine whether this effect is recapitulated *in vivo*. U251 GBM cells were transfected with si-uc. 110-1 or si-uc.110-2. After 2 days, these cells were implanted into immunodeficient mice using intracranial injection (Supplementary Figure 9). [37, 38, 45, 46] Tumor growth was monitored by MRI and mouse survival was observed over a period of 70 days. Mice that were xenografted with U251 cells that were transfected with si-uc. 110-1 and si-uc.110-2 expression developed smaller tumors, as depicted, and quantified by MRI (Figure 5A, 5B). The mice that received si-uc.110 also displayed better overall survival than mice that received scrambled control siRNA cells (Figure 5C).

### uc.110 regulates the expression of the Wnt pathway member, Membrane Frizzled Related Protein (MFRP).

**Figure 6.**
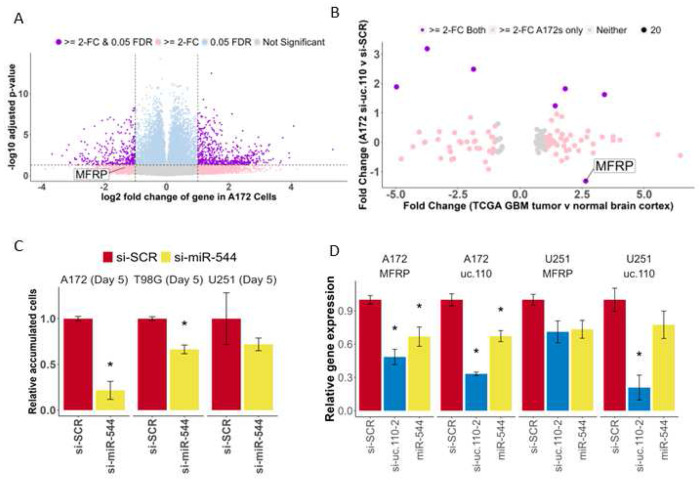
The uc.110 TUCR activates Wnt-signaling by sponging miR-544 from membrane frizzled related protein (MFRP) 3’UTR. A) Volcano plot depicting transcriptome deregulation in RNA-Seq data on A172 GBM cells transfected with si-uc.110. Purple dots represent genes that are significantly deregulated >= 2-fold. Blue dots represent genes that are significantly deregulated. Pink dots represent genes that are deregulated >= 2-fold. Gray dots are neither deregulated nor significant. B) Dot plot showing that, of the genes that are predicted miR-544 targets, MFRP is the only gene that is upregulated in GBM Tumors and downregulated when uc.110 is downregulated. Purple dots represent predicted miR-544 targets that are deregulated in A172 cells from 6A and TCGA RNA-Seq data. Pink dots represent predicted miR-544 targets that are deregulated in A172s from 6A only. C) Bar graph showing that miR-544 transfection reduces cell accumulation in A172, T98G, and U251 cells, confirming its tumor suppressor role. Facets represent cell lines. si-SCR = scrambled control siRNA (red), si-uc. 110-2 = siRNA targeting uc.110 at nucleotide 195/243 (blue). miR-544 = miR-544 (yellow). D) Bar graph showing that transfection with miR-544 or si-uc.110-2 reduces uc.110 and MFRP expression. Facets represent genes and cell lines. * = p < 0.05

LncRNAs can have various functions that depend on their subcellular localization. Nuclear lncRNAs are usually involved in transcriptional regulation, while cytosolic lncRNAs are usually involved in translational and spatial regulation. [2] We fractioned four GBM cell lines (A172, U251, U87, U1242) into nuclear and cytosolic fractions. When compared to nuclear (U44, U48) and cytosolic (GADPH, PPIA) controls, uc.110 appears to be localized to both the nucleus (mainly in U87, U251, and U1242 cells), and the cytoplasm (mainly in A172 cells) (Supplementary Figure 8G). We then performed RNA-Seq on A172 cells that had been transfected with si-SCR, si-uc.110-1, or si-uc.110-2 for 48 hrs. and found several genes that are deregulated when uc.110 expression is downregulated (Figure 6A). To identify genes that are particularly related to uc.110 function, we focused on genes that demonstrated coregulation with uc.110 in our WGCNA analysis (Figure 3E). Of particular interest was the membrane frizzled related protein, also known as MFRP. [47, 48] MFRP serves as a shuttle for the Wnt-ligand, and functions as an activator of the Wnt-signaling pathway. This gene was the only gene in our analysis that correlated with uc.110 expression, was upregulated in GBM tumors, and downregulated when uc.110 is knocked down in A172 cells, suggesting MFRP coregulation with uc.110. (Figure 6B).

### uc.110 sponges the tumor suppressor microRNA miR-544 to increase the bioavailability of MFRP and WNT activity in GBM.

**Figure 7.**
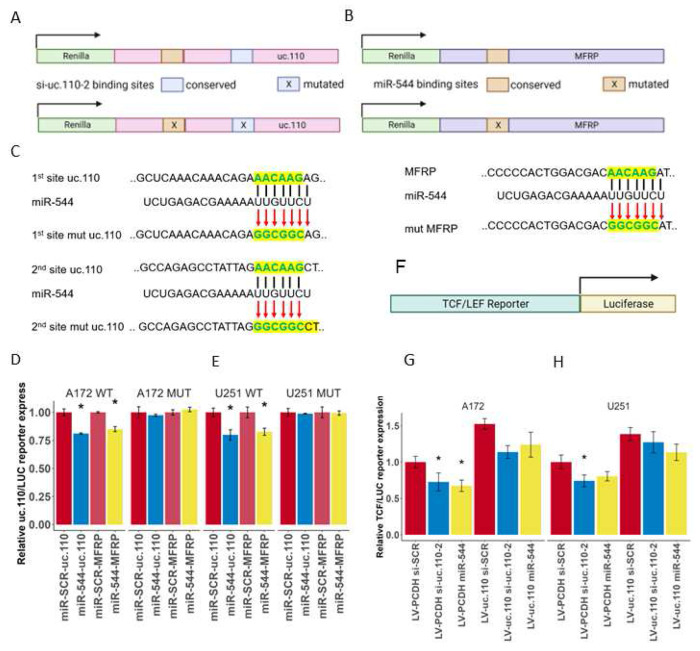
The uc.110 TUCR activates Wnt-signaling by sponging miR-544 from membrane frizzled related protein (MFRP) 3’UTR. A) Schematic depicting the uc.110 luciferase construct used to demonstrate binding to miR-544 and si-uc.110-2. Binding of miR-544 to binding sites (orange) leads to a degradation of construct and a reduction in Renilla signal (Green) B) Schematic depicting the MFRP luciferase construct used to demonstrate binding miR-544. C) Schematic depicting miR-544 binding site mutations for uc.110 (two sites) and MFRP (one site). Top row represents wild-type binding sites. Middle row is the miR-544 binding region. Bottom row are mutated sites. D) Bar graph showing that transfection of miR-544 reduces uc.110 and MFRP luciferase expression signal in A172 and E) U251 glioma cells, and that mutating miR-544 binding sites rescues luciferase signal. Facets represent cell lines and miR-544 binding site mutation status. F) Schematic depiction of TCF/LEF luciferase reporter construct used to measure downstream Wnt-signaling pathway activation. TCF/LEF binds to the reporter region (green) and activates luciferase (yellow). F) Bar graph showing that transfection of si-uc.110-2 and miR-544 reduces TCF/LEF reporter signal in A172 and H) U251 glioma cells. Signal is rescued when uc.110 is overexpressed in the presence of siRNA or miR-544. * = p< 0.05 the letters.

One common lncRNA mechanism of action is as a miRNA sponge, acting as a binding competitor for various miRNAs and therefore increasing the bioavailability of those miRNAs’ targets. [2, 49, 52–53] Based on the WGCNA data that we generated above, we hypothesized that uc.110 may function by sponging miRNAs away from MFRP transcripts, as their expression relationship is consistent with such an interaction. We hypothesized that a tumor suppressor miRNA can successfully target and suppress MFRP in the normal brain (Supplementary Figure 10A). This leads to downstream activation of Wnt target genes involved in biological processes such as cell accumulation, invasion, and stem cell differentiation (Supplementary Figure 10B). We further hypothesized that in glioma tumors, uc.110 is activated and acts as a binding competitor for this miRNA (Supplementary Figure 10C), increasing the bioavailability of MFRP and increasing Wnt pathway signaling (Supplementary Figure 10D). To identify candidate miRNAs that are consistent with the afore mentioned hypothesis, we screened public databases and published literature for GBM tumor suppressor miRNAs that are predicted to bind to both uc.110 and MFRP. The only miRNA that fulfilled these criteria was miR-544. We first investigated the functional effects of miR-544 in GBM cells. Transfection of miR-544 into U251, A172, and T98G GBM cell lines reduced cell accumulation after 5 days (Figure 6C). Expression of both uc.110 and MFRP in GBM cells was reduced when transfected with miR-544 or si-uc.110 (Figure 6D). To further test the hypotheses, we asked if miR-544 targets both uc.110 and MFRP, and if this binding affects Wnt signaling. To determine whether MFRP and uc.110 are direct targets of miR-544, we constructed luciferase reporter vectors by inserting the uc.110 ultraconserved region and MFRP 3’UTR downstream of hRluc followed by Synthetic Poly(A) using psiCHECK-2 backbone vector (Promega) (Figure 7). We first measured target binding by transfecting the reporter constructs followed by transfection with miR-544 or miR-SCR (scrambled control) in GBM cells. Ectopic expression of miR-544 significantly decreased luciferase activity compared to miR-SCR (Figure 7D, left panel and figure 7E, left panel). These binding sites for miR-544 were predicted via computational algorithms and validated via sequencing. We then mutated the binding sites for MFRP and uc.110 (Supplementary Figure 10, Figure 7C) and assessed signal strength again. The data showed that luciferase activity was not significantly altered in mutant-reporter-vectors transfected cells (Figure 7D, right panel and figure 7E, right panel), indicating that miR-544 binds to both uc.110 and MFRP in GBM cells, and that this binding is lost when the miRNA binding sites are mutated.

Lastly, we asked if uc.110 expression alters Wnt pathway activity. To answer this question, we studied one of the most established downstream targets of Wnt-signaling, the T cell factor/lymphoid enhancer factor family (TCF/LEF). When Wnt-signaling is activated, TCF/LEF is produced downstream and activates Wnt-signaling target genes. Therefore, TCF/LEF activity can be used as a proxy for pathway activity and can be measured with a TCF/LEF luciferase reporter assay. (Figure 7F). The activity of this reporter can be regulated by either directly reducing Wnt bioavailability with miR-544 or indirectly by targeting uc.110 with siRNA. If upstream Wnt signaling is reduced, the luciferase construct will bind fewer activators and exhibit decreased signal. Likewise, we would expect that overexpression of uc.110 would rescue the bioavailability of MFRP and consequently also downstream activation of the TCF/LEF construct. We found that transfection of A172 and U251 cells with si-uc.110 and miR-544 reduced reporter activity in A172 (Figure 7G) and U251 (Figure 7H) cells, and that this effect can be rescued via uc.110 overexpression. These data taken in conjunction provide strong support for a miRNA sponge model for the uc.110 oncogene. Altogether, the above data demonstrate an important role for uc.110 in regulating the Wnt pathway in GBM by sponging the Wnt inhibitory miRNA miR-544 (model shown in Supplementary Figure 10).

## DISCUSSION

This study investigated Transcribed Ultraconserved Regions (TUCRs), a set that might contain long noncoding RNA sequences that are fully conserved across human, mouse, and rat genomes. These TUCRs are distinct due to their exceptional conservation, which often signifies functional importance. Despite their potential significance, TUCRs have been minimally explored, especially in relation to cancer. Of note, the findings of this study represent the first of their kind on TUCRs in gliomas and the first comprehensive analysis of TUCR expressions and functions in any cancer. They contribute critical new insights into an uncharted area of glioma biology, while also providing a novel framework for studying TUCRs in other cancers and other diseases, where they are also understudied.

We found that TUCRs are located across the genome, resistant to variation, and actively transcribed. We manually annotated each as either exonic, intronic, exonic/intronic, or intergenic. We identified distinct signatures for intergenic and intragenic (exonic, intronic, exonic/intronic) RNAs. Intragenic TUCRs are expressed at a level that is most like coding genes, while intergenic TUCRs more closely resemble lncRNAs. We then performed the first analysis of TUCR expression in gliomas and found that the majority of TUCRs are deregulated >= 2-fold in GBM and LGG, with a 56% overlap. This shows that TUCRs are not only expressed, but also frequently dysregulated in gliomas compared to normal brain tissue. This is critical, as their high degree of conservation and dysregulation suggests that they may serve critical biological functions. We then extended our analysis to TUCR correlation with patient survival. In GBM, the extremely short survival times (15 months) limit the detection of significant correlations. However, patients with LGG live substantially longer (84 months), and therefore more TUCRs are associated with patient outcomes in this disease, suggesting a potential impact on glioma patients’ prognoses and indicating possible novel biomarkers. Another facet of our research involved predicting the functions and mechanisms of action of TUCRs in gliomas. We studied this for the first time in gliomas WGCNA workflows to cluster TUCRs and provide functional predictions based on shared functions between coregulated genes. This approach identifies a wide range of potential functions for TUCRs, encompassing activities such as nucleic acid binding regulation, stem cell differentiation, organ development, immune response, and cell signaling.

We found intergenic TUCRs to be of notable interest because they resemble lncRNAs but are much more highly conserved and experience less sequence variation. Notably, these TUCRs do not overlap with known genes, suggesting they might represent novel lncRNAs. Of these TUCRs, uc.110 is the most upregulated in both GBM and LGG. Knocking down uc.110 reduces cancer cell characteristics *in vitro* and *in vivo* and improves survival in mouse models. On the other hand, increasing uc.110 expression increases malignancy in cells that do not express it, further indicating its potential oncogenic role. We explored uc.110’s function via WGCNA, revealing its membership in modules associated with oncogenic nucleic acid binding. We integrated these data with transcriptome deregulation data (RNA-Seq) post-uc.110 knockdown, revealing a close relationship between uc.110 and the oncogenic membrane frizzled-related protein (MFRP). This protein is involved in activating the Wnt-signaling pathway, impacting cell proliferation, invasion, migration, and stem cell differentiation. From these data we hypothesized that uc.110 might sponge tumor suppressor miRNAs from MFRP, enhancing Wnt signaling activation. Accordingly, we demonstrated that one mechanism of action for the uc.110 oncogene is as a miRNA sponge for miR-544, therefore increasing the bioavailability of MFRP and Wnt activation.

In conclusion, our results indicate that TUCRs are an important class of regulatory RNAs. They are more highly conserved than typical genes and more resistant to variation, which suggests biological importance. They are perturbed in gliomas, and this perturbation is associated with clinical outcomes. Our predicted functions reveal that TUCRs are widely involved in cancer-related biological processes. Some TUCRs previously thought to be intergenic may represent previously undiscovered genes. Our findings also identify and characterize uc.110 as a new oncogene in gliomas. Each of the experiments performed in our study represents the first of its kind in gliomas. We have developed, adapted, and presented novel methods for studying TUCRs that can be used in other cancers and other diseases, where TUCRs remain very understudied. These methods and the data derived from them represent a “TUCR database” that will serve the scientific community in future TUCR studies in gliomas and other diseases, where they remain unstudied or understudied.

## MATERIALS AND METHODS

### Data Availability Statement

RNA-Seq data for Figure 6A will be made available on the Gene Expression Omnibus (GEO) prior to publication. Detailed TUCR results can be found at http://www.abounaderlab.org/tucr-database/. Please refer to the corresponding author for any data access questions.

### Detailed Computational Methodologies

Detailed methods, including access to information for all datasets used, can be found in a repository at: github.com/abounaderlab/tucr_project.

### TUCR Annotations [29, 30]

TUCR annotations were performed manually by overlaying consensus TUCR genomic annotation tracks to the hg38 human genome in the UCSC Genome Browser. In parallel, bedtools closest was used to identify genes that are intergenic or intragenic. These results were then cross referenced to identify a consensus genomic annotation for each TUCR. Detailed methods can be found at github.com/abounaderlab/tucr_project

### TUCR Chromatin Landscaping

U87 H3K4me3, RNA Pol.II, and H3K27ac CHIP-Seq data and U87 ATAC-Seq data were acquired from the Gene Expression Omnibus. Randomized control TUCRs were generated using Quinlan Labs’ bedtools [31, 32] and the shuffle command.[31, 32] Bedtools fisher and R/RStudio [53, 54] were used to perform chi-square tests to compare predicted overlaps of peaks to expected peaks. Detailed methods can be found at github.com/abounaderlab/tucr_project

### TCGA AND GTEx RNA-Seq Data [33, 34]

GBM (n = 161) and LGG (n = 505) RNA-Seq data were acquired from the Cancer Genome Atlas and were compared to normal brain cortex from the Genotype-Tissue Expression Database (GTEx, n = 260) using a workflow including bedtools, bowtie, the SRA toolkit, and R/RStudio. Detailed methods can be found at github.com/abounaderlab/tucr_project

### TUCR Expression, Deregulation, and Survival Analyses [33, 34, 54, 55]

TUCR expression, deregulation, and survival analyses, were analyzed using processed TCGA and GTEx RNA-Seq data and a workflow using R/RStudio. Detailed methods can be found at github.com/abounaderlab/tucr_project

### TUCR weighted gene correlation network analysis (WGCNA) [36]

TUCR WGCNA was performed using processed TCGA and GTEx RNA-Seq data using a modified version of the R/RStudio workflow designed by Drs. Peter Langfelder and Steve Horvath at UC Los Angeles. Detailed methods can be found at github.com/abounaderlab/tucr_project

### De novo transcript reassembly and validation [35]

De novo transcript assembly was performed on TCGA GBM and LGG RNA-Seq data using standard protocols and the *stringtie* bioinformatics package. Results were validated using PCR: 10 min at 95°C, followed by 40 cycles of 10 seconds at 95°C and 1 minute at 60°C. Detailed methods can be found at github.com/abounaderlab/tucr_project.

### Patient Samples

GBM Tumor samples were acquired from the UVA Tumor Bank. Detailed patient information can be found as a supplement (UVATumorBank_data.csv).

### Cell Lines and stem cells

U87, U251, A172, and T98G glioblastoma cell lines were used in *in vitro* experiments and were acquired from ATCC. U87 cells were cultured in 500 mL minimum essential media (MEM) Earles (Gibco, #.11095-080) containing 5 mL penicillin/streptomycin (pen/strep, Gibco, Cat #.15140-133), 5 mL MEM non-essential amino acids (NEAA, Gibco, #.11140-050), 5 mL sodium pyruvate (Gibco, 100 nM, #.11360-070), 10 mL sodium bicarbonate (Gibco, 7.5%, #.25080-094), and 50 mL fetal bovine serum (FBS). T98G cells were cultured in 500 mL MEM Earles media containing 5 mL pen/strep, 5 mL NEAA, 5 mL sodium pyruvate, and 50 mL FBS. A172 cells were cultured in 500 mL Dulbecco’s modified eagle media (DMEM, Gibco, #.11965-092) containing 5 mL pen/strep, and 50 mL FBS. U251 cells were cultured in 500 mL RPMI L-Glutamine media (Gibco, #.11875093) containing 5 mL pen/strep and 25 mL FBS. GSC-34 and GSC-28 glioblastoma stem cells were cultured in neurobasal (L-glutamine negative) media (Gibco, #.21103-049) containing 5 mL pen/strep, 5 mL B-27 (without Vit-A, Gibco, #.12587-010), 2.5 mL N-2 (Gibco, #.17502-048), 1 mL EGF, 1 mL FGF, and 1.25 mL L-Glutamine. All cell media contained in 5 μL Plasmocure reagent to prevent mycoplasma contamination.

### Primer and Oligo Design

Primers and siRNAs were designed using the Primer3 and Thermofisher design portals respectively. uc.110 forward primer sequence is 5’-CAGCCAAAGGGGAAGTGTAT-3’, and the reverse sequence is 5’-CCGTCCTCCCTGCACTAAAT-3’.

MFRP forward primer sequence is 5’-GCATCTATTCATGTGGCAGGC-3’, and the reverse sequence is 5’-TACTCCGGACCCTCCAGTTG-3’.

The miR-544 precursor was ordered from Invitrogen (#.AM17100). Negative control oligos were ordered from Ambion (#.AM4635).

### uc.110 stable overexpression

The full uc.110 transcript from “de novo transcript reassembly and validation” was cloned into the pCDH-EF1-MCS-BGH-PGK-GFP-T2A-Puro vector (# CD550A-1) using stbl3 competent E. coli cells and ampicillin selection. Amplified vector was extracted using the miniprep kit (Qiagen, #.27106). 0.75 μg of this vector, 0.75 μg of psPAX2 lentiviral gag-pol packaging vector, and 0.5 μg of pMD.2G VSV-G enveloping protein was transfected in 6 μL X-tremeGENE transfection reagent (#.06366236001) into 293T cells per manufacturer instructions to generate a lentivirus that was transduced to U87, U251, and A172 cells in media without antibiotics. These cells were subjected to antibody (puromycin) selection for uc.110-positive cells at D3.

### uc.110 quantitative (q)PCR

Total RNA was isolated using the RNEasy+ kit (Qiagen, #.74134) according to manufacturer instructions. RNA concentration and purity was measured via nanodrop. 800 ng of cDNA was synthesized (BIORAD T100 Thermal Cycler) using the iScript (BIORAD, #. 1708890) synthesis kit per manufacturer instructions. A 20 μL reaction mixture was then created for each condition with the following concentrations: 1 μL of combined forward/reverse primers (5 μM), 10 μL of iQ SYBR Green master mix (#1798880), 4 μL of nuclease free water, and 5 μL of synthesized cDNA. These reactions were cycled (BIORAD CFX Real Time System) in 96-well plates: 10 min at 95°C, followed by 40 cycles of 10 seconds at 95°C and 1 minute at 60°C.

### Cell Counting (Accumulation) Assay [37–39, 44]

Cells were seeded in 6-well culture plates with full serum media at 30,000/well density at D-1. At D0, each well was transfected via master mix 3 μL of siRNAs (20 μM) via 9 μL Lipofectamine 2000 (Invitrogen, #.11668-019) in 300 μL OPTI-MEM (Gibco, #.31985-070) and 700 μL antibiotic and empty media for 6 hours. At 6 hours, media were replaced with fresh media containing antibiotics and FBS. Cells were then counted via haemocytometer at Days 1, 3, 5, and 7 for each cell line.

### Transwell Cell Invasion/Migration Assay [42–44]

Cells were seeded in 6-well culture plates with full serum media at 300k/well density at D-1. At D0, each well was transfected via master mix 3 μL of siRNAs (20 μM) via 9 μL Lipofectamine 2000 in 300 μL OPTI-MEM and 700 μL antibiotic and empty media for 6 hours. At 6 hours, the media were replaced with fresh media containing antibiotics and FBS. The cells were then seeded in empty media at 200k/chamber into Transwell Invasion Chambers coated with Collagen IV. After 8 hours, non-invading cells were cleared and invading cells were stained with Crystal Violet.

### AlamarBlue Cell Viability Assay [40–41]

Cells were seeded in 96-well culture plates with full serum media at 10k/well density at D-1. Border wells were filled with media to account for edge effects. At D0, each well was transfected via master mix 1 μL of siRNAs (20 μM) via 3 μL Lipofectamine 2000 in 30 μL OPTI-MEM and 70 μL antibiotic and empty media for 6 hours. At 6 hours, media were replaced with fresh media containing antibiotics and FBS. Functional assays were performed using the AlamarBlue kit (Life Technologies #. A50100) per manufacturer instructions. Reactions were allowed to proceed for 1 hour.

### Ex vivo knockdown of uc.110

Cells were seeded in 6-well culture plates with full serum media at 300k/well density at D-1. At D0, each well was transfected with 3 μL of siRNAs (20 μM) via 9 μL Lipofectamine 2000 in 300 μL OPTI-MEM and 700 μL antibiotic and empty media for 6 hours. At 6 hours, the media were replaced with fresh media containing antibiotics and FBS. Mouse experiments were performed using xenograft models and intracranial injections of U251 cells post transfection with siRNA oligonucleotides. Cells were injected at D2 and were imaged at two-week intervals via MRI. Survival was assessed sured daily and tumor volume was measured at the end of life.

### RNA-seq post-uc.110 knockdown

Cells were seeded in 6-well culture plates with full serum media at 300k/well density at D1. At D0, each well was transfected with 3 μL of siRNAs (20 μM) using 9 μL Lipofectamine 2000 in 300 μL OPTI-MEM and 700 μL antibiotic and empty media for 6 hours. At 6 hours, the media were replaced with fresh media containing antibiotics and FBS. RNA Libraries were collected and sequenced via RNA-Seq on Day 2 (post transfection).

### Luciferase Reporter Vector Construction

The Luciferase reporter vector were constructed via insertion of uc.110 conserved region and 3’UTR of MFRP downstream of Renilla luciferase stop codon in psi-CHECK2 dual luciferase vectors (Promega, Madison, WI, USA). The insertions were validated by sequencing. Uc.110 and MFRP primer pairs with XholI and NotI sequence at 5’ and 3’ respectively, uc.110-FW: 5’-ATATATctcgagCGAGGTGAGAACCAGAGTGT-3’, uc.110-RW: 5’-AATAATgcggccgcTTGGCTGCCTAATGAGTCACA-3’, MFRP-FW: 5’-ATATATctcgagAAATGGGGTCTGGTCCTTGG-3’ and MFRP-RW: 5’-AATAATgcggccgcTCGCCTTTCTCTCCCGGA-3’ were used for PCR amplification. Site-directed mutagenesis of predicted miR-544 target sites for both uc.110 and MFRP were performed to generate mutant vectors.

### 3’UTR Reporter Assays

To determine whether miR-544 directly binds to the MFRP 3’UTR and uc.110, cells were transfected with miR-544 or miR-scr (control) for 24 hour. The cells were then transfected with luciferase reporter control or 3’UTR-MFRP or uc.110 as well as corresponsive mutant vectors for 24 hours. Luciferase assays wered performed using the Luciferase System Kit (Promega) and luminescence was measured. Renilla luciferase activity was double normalized by dividing each well first by firefly activity and then by average luciferase/firefly value in a parallel set done with constitutive luciferase plasmid.

### TCF/LEF reporter Assays

Cells were seeded in 6-well culture plates with full serum media at 300k/well density at D-1. At D0, each well was transfected with 3 μL of siRNA/miRNA (20 μM) using 9 μL Lipofectamine 2000 in 300 μL OPTI-MEM and 700 μL antibiotic and empty media for 6 hours. At 6 hours, the media were replaced with fresh media containing antibiotics and FBS. MFRP and uc.110 sequences were cloned into the PROMEGA pmirGLO Luciferase vector (E1330). BPS Dual reporter luciferase assays were ordered for TCF/LEF (#.60500) and uc.110/MFRP (#.60683) experiments.

### In Vivo Tumor Formation

Adult male and female Nude: Hsd:Athymic Nude-Foxn1 mice were purchased from Harlan. All the animal work was conducted at the Animal Research Core Facility at the University of Virginia School of Medicine in accordance with the institutional guidelines. Mice used for this study were anesthetized with ketamine (17.4 mg/20g), xylazine (2.6 mg/20g) and placed on a sterotactic frame. Tumor xenografts were generated by implantation of U251 cells transfected with si-uc. 110-1, si-cu.110-2 or si-Scr. U251 cells (3x10^5^ cells; n=5) were stereotactically implanted into mice in their right striata at the coordinates from the bregma 1mm anterior, 1.5 mm lateral and 2.5 mm intraparenchymal. Three weeks after tumor implantation, the animals were subjected to brain MRI. To measure the tumor size, 20 ul of gadopentetate dimeglumine (Magnevist, Bayer Healthcare) was intraperitoneally injected 15 minutes before scanning. Tumor volumes were measured using MicroDicom.

### Statistical Analyses

Comparisons between means of samples were performed using Student’s t-test and one-way ANOVAs. Comparisons between categorical variables were performed using chi-squared and Fisher’s exact test. Comparisons were considered statistically significant if the p-value was less than 0.05. Molecular experiment tests were performed in SigmaPlot 14.0, while computational experiment tests were performed using bedtools and/or RStudio. Detailed methods can be found at github.com/abounaderlab/tucr_project

## Supplementary Material

1
