# Supplementary material for: A first comprehensive analysis of Transcribed Ultra Conserved Regions uncovers important regulatory functions of novel non-coding transcripts in gliomas
Source: Res Sq. 2024 Apr 18:rs.3.rs-4164642. Preprint. [Version 1] doi: 10.21203/rs.3.rs-4164642/v1 (PMC11065071; doi:10.21203/rs.3.rs-4164642/v1)
Supplement: 1 [file NIHPPrs4164642v1-supplement-1.pdf]

bioRxiv preprint doi: https://doi.org/10.1101/2023.09.12.557444; this version posted March 20, 2024. The copyright holder for this preprint (which was not certified by peer review) is the author/funder. All rights reserved. No reuse allowed without permission.

SUPPLEMENTARY DATA

Supplementary Figure 1

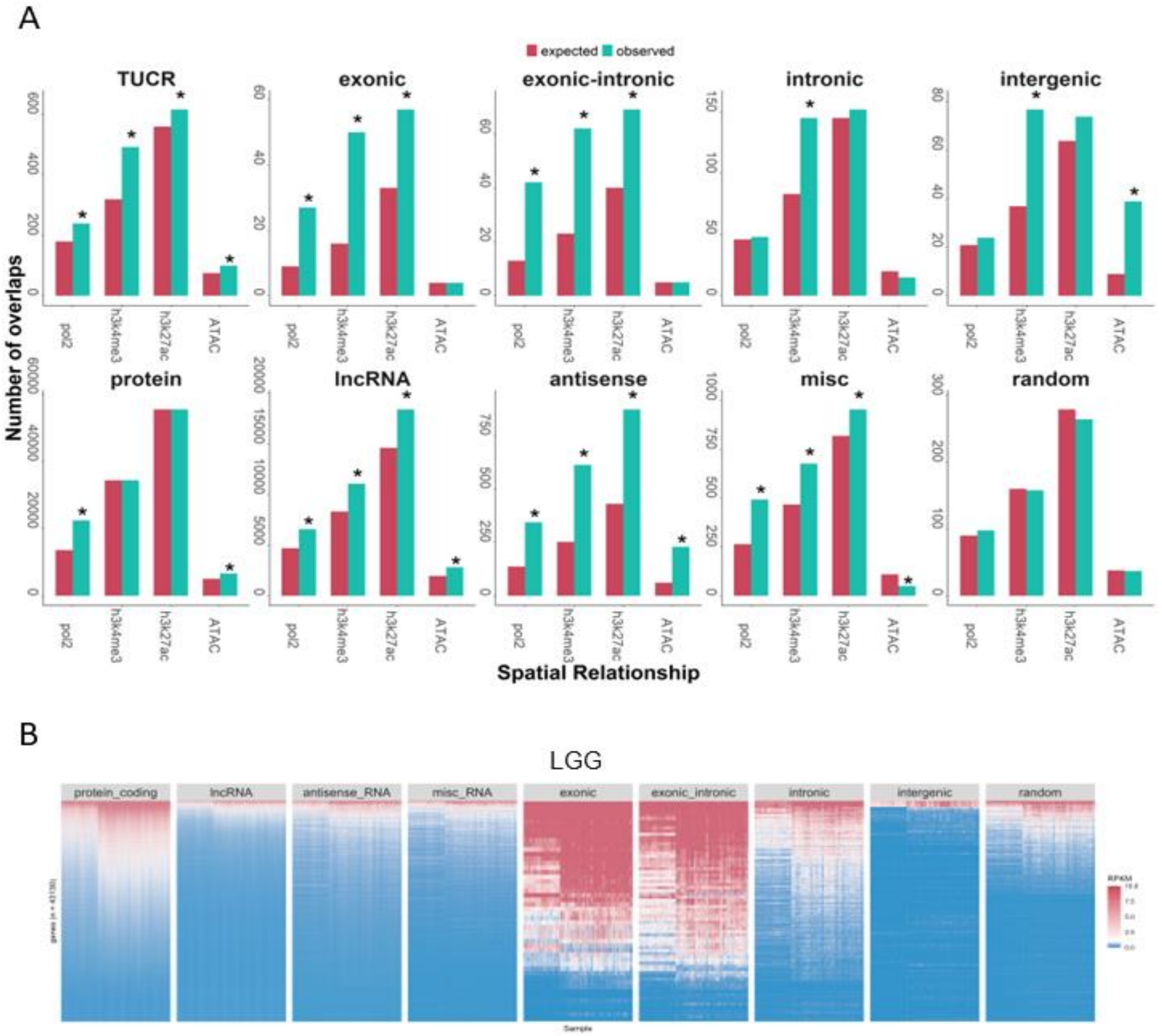

*<u>Supplementary Figure 1. Annotation, localization, and expression of TUCRs in GBM and LGG.</u> A) Bar chart showing that TUCRs are enriched for markers for open and active chromatin in GBMU87 cells, suggesting that they represent transcriptionally active sites. Red bars represent chi-square expected overlaps, and teal bars represent observed values. B) Heatmap representing TUCR absolute expression (RPKM) across multiple gene annotations. Blue represents poorly expressed genes (<1 RPKM), White/Pink genes are moderately expressed (>=1 RPKM) and Red represents highly expressed genes (RPKM >=10). TUCRs demonstrate an expression profile that is comparable with protein coding genes. * = p < 0.05*

bioRxiv preprint doi: https://doi.org/10.1101/2023.09.12.557444; this version posted March 20, 2024. The copyright holder for this preprint (which was not certified by peer review) is the author/funder. All rights reserved. No reuse allowed without permission.

Supplementary Figure 2

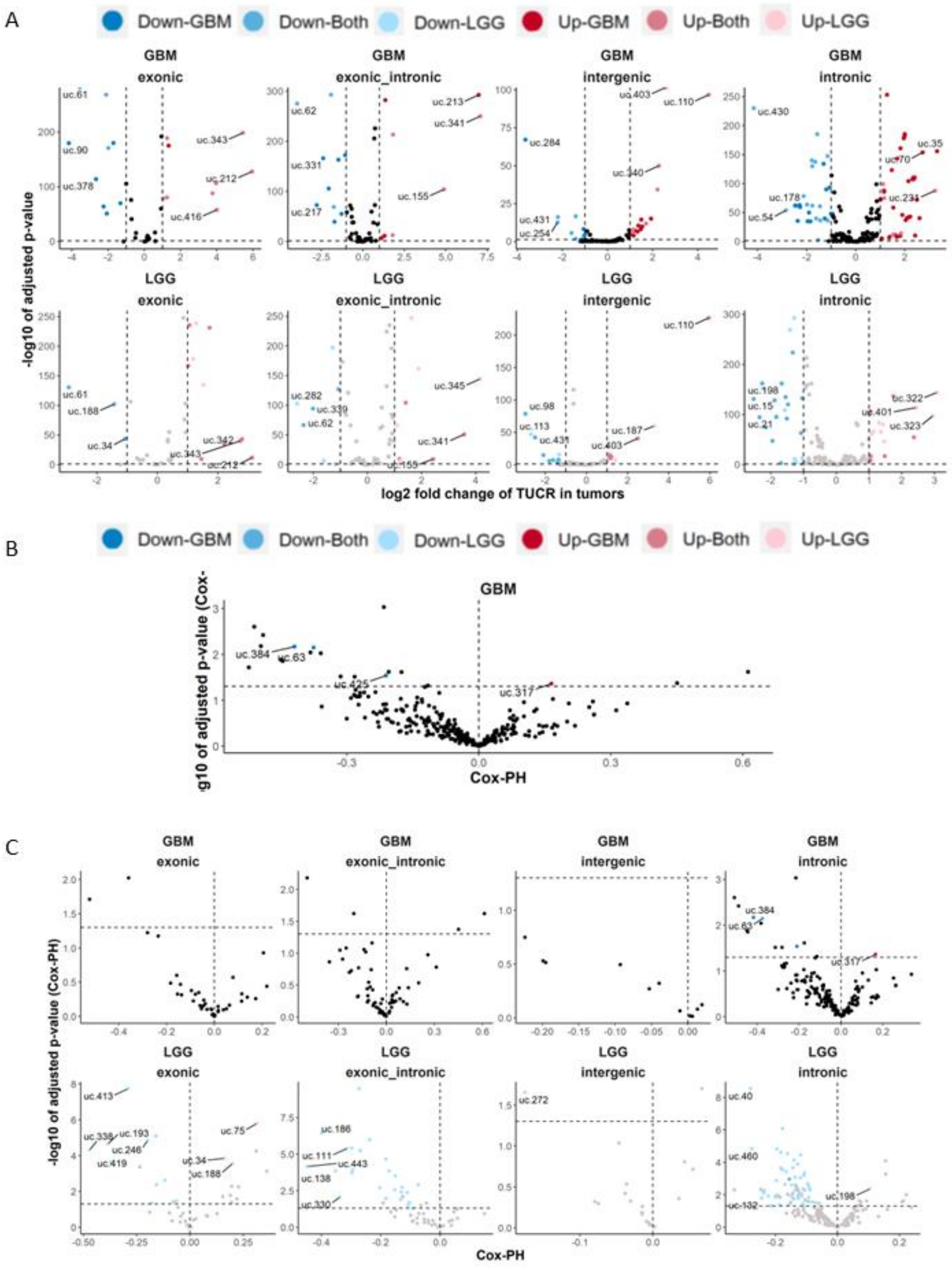

Supplementary Figure 2. TUCRs are deregulated and associated with patient outcomes in gliomas. *All experiments were performed using TCGA GBM and LGG RNA-Seq data. A) Volcano plots showing that TUCRs are deregulated in every TUCR annotation category in GBM and LGG. Red dots are upregulated. Blue are downregulated. B) Volcano plot showing that few TUCRs are significantly associated with patient outcomes in GBM. Red dots represent TUCRs significantly associated with poor prognosis. Blue dots represent TUCRs significantly associated with good prognosis. C) Volcano plot showing that TUCRs in every TUCR annotation category are associated with survival in gliomas. Red dots represent TUCRs significantly associated with poor prognosis. Blue dots represent TUCRs significantly associated with good prognosis.*

bioRxiv preprint doi: https://doi.org/10.1101/2023.09.12.557444; this version posted March 20, 2024. The copyright holder for this preprint (which was not certified by peer review) is the author/funder. All rights reserved. No reuse allowed without permission.

Supplementary Figure 3

uc.110

A

Chrom	Start	End	Length	ID
chr2	236,162,737	236,162,980	243	uc.110
chr2	236,161,295	236,164,636	3341	TUC110

B

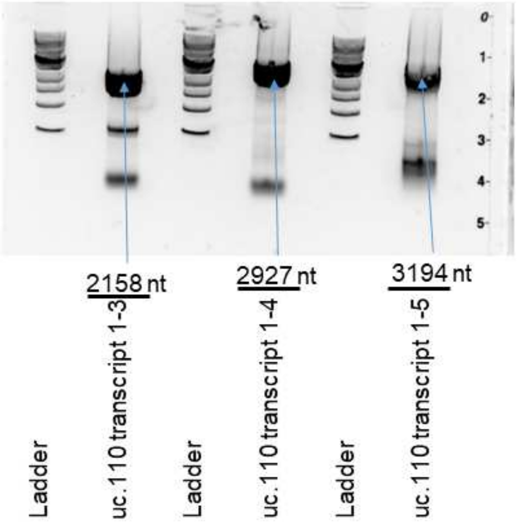

C

```
TCGGCTTAGGCCCCCACTTCTGAACTGTTCAAATAGACAATCGGTTTTGATGATGTAATTTATAATTTATGCAGGTT
GTCTACATACATATGGTGTTGCTTATGTGCGTTGGGGTTTCAGGTGCTTTATTACTCTGCCGGTGTGATTTGCATTC
ATGTTTGGTTACCAAGATCATATTTCTCCTGTTTACACCCTAAGGGCCTAAAATGAAGATGAATGCCACCTCCCTTC
GTCTGGGCTGTCCCTGTAGAACCCCGGCCTTAGCTTCCCCCAAGACCCCAGAGCCCTGCTACCCTGGAACTGGGCC
CTGAGCACTGCGCTGAAGCTGCCTGGGAGGACCCAGCGGCCAGAAGGTCGGAGCAGGCCACCTGCTCTGGCTGT
GCATGCCTGGGCAGATGCCTGGGGTCTCTGGGGACAGAAATAGGTGTGTGCACTGGAGCCTCCTTCCCTGGGAA
GCCACGGCTGGGCATAAGCGAGCCACTCTGCCACACCGCCACTGAAGTCCAGCAGGGGAAAGAAGAGGGCCCCG
AGAGGGTCTCTGCTGCCCGCGCTGCTGAATGCGTGGGCTCCAGCCACTTGGGACTAGGAAGGCCCCGAGGTGAG
AACCAGAGTGTGATCCAAAGGCTCCTGAGGCCGGAAAGCTAGAAGGTCACTCCCGGCTTTCAGTGCACCTAGAAC
CACCCGCCATGGGGCATAACATGGGACTCTGTTCCAGACAGGTCTGGGGCTGGAGGGAGCAGCCTTGGAAGCCC
TGTGCGAGCTTCACCAGATGCAGCTGAAATAATTAAGCCCATTAAGCTCAAACAAACAGAAACAAGAGGGAGAG
GGTGCTGACCCTGAGAGAAAGGGTGTCTTGGAATGCAGGTACCAGCGATGTGCCCAGGCTTGTACAAGCACCTA
GGCTGAAGGCCAGACGCACAGACAGCCATAGAGTCAGGATGTCCTCCGAGGCAAACCCGGGCTCAAAAAAGCCT
GGGGGGGCCACTCCCTGCTCTCCATTTAGCTGACAAGGCATCTGTGAAAGAACCACCCAGGGGGCTGAAGACAG
GAAGCAAAGGCTGGATGCACAAGCATGCACTAAGTCTCGAAAATTCCAGCGCAGGTTGGGATTTGAGCAGCTTCC
AGTGCAGGTGGGTCCCGGCTGCTGGGAAGGTAGTCCTGGGCTCCTTCTGGAAGGGGAAGTTGCTTGAGGCCCAG
GACCAAACTAAAGAGGGTCCTGCCCCAAGAACAGGGAGATGGCTGCGGATTTTCAGCATACCTTGGGTTCTCCAA
TGGGGCAGGGGTGCCTGGCTTGCCAGGGAGCAAAGGCAGTCACTGGGTGGAGTGACTGTTCTTGTGGCCCCCCT
CCGACCTCTGCCTGGGTCGGTGGCTGCGGGGTGCAGGCAGCCCTGAGGCGTCCCAGTGCACGGCTGGGGGGCGC
AGGTCCAAGGAGCTCCCAAGTGTAATTATTTGATctgcagatgtaagtggaatttattgtaacaacaaatatagtgatgtcaaaccc
aaccttggattaaagccggcagccaaaggggaagtgtattaatttccaacagcatctcaggccagagcctattagaacaagctattcttcaggccccc
ccatcagaattaatcattgggagttaatttgaagctcagacaagttgctgttaatttagtgcagggaggacgggatggaataaaaagctgaggtgctg
gccgagcctcgtgggtctcattaatcagccagctgagacggcctggcttgattacaatttgcaaaaaaattcattagggcccgggcgattgacaattttt
ctctctgcctgtgatgtgactcattaggcagccaaatgaaagccatgatgacggccatgatgacagcggccatcaagcgctgcttgtttatcttcctccc
ctcggcctgtctcccgggccttggcctccctgccgggctctcgggggcgccccggggctcaggggttagggagggagggggtcagcgccccgaggag
caggagggagggcggcatcggccagcttggggagctcgggccctctcctcctcgtgcctccatagggcgaggacctcgaggctgggagggcccgga
gctcggtcccaggagcggggcttcctgacggcggcctccggttcctagtgggcttcgcttt
```

Supplementary Figure 3. Elucidation of the uc.110 TUCR full transcript sequence. *A) We used de novo transcript reassembly of TCGA glioma RNA-Seq data and experimental PCR validation to identify the predicted sequence for the novel full RNA transcript containing uc.110. Table depicts uc.110 ultraconserved and predicted full transcript genomic locations and length in nucleotides (nt). B) PCR gel electrophoresis demonstrating validated uc.110 transcript variants. C) The validated full sequence of the 2,158 nt uc.110 transcript is provided. The ultraconserved uc.110 region is colored red and primer sequences are colored green. The additional variants (1-4 and 1-5) were also validated but de-emphasized due to variant 1-3 having the strongest functional effect.*

bioRxiv preprint doi: https://doi.org/10.1101/2023.09.12.557444; this version posted March 20, 2024. The copyright holder for this preprint (which was not certified by peer review) is the author/funder. All rights reserved. No reuse allowed without permission.

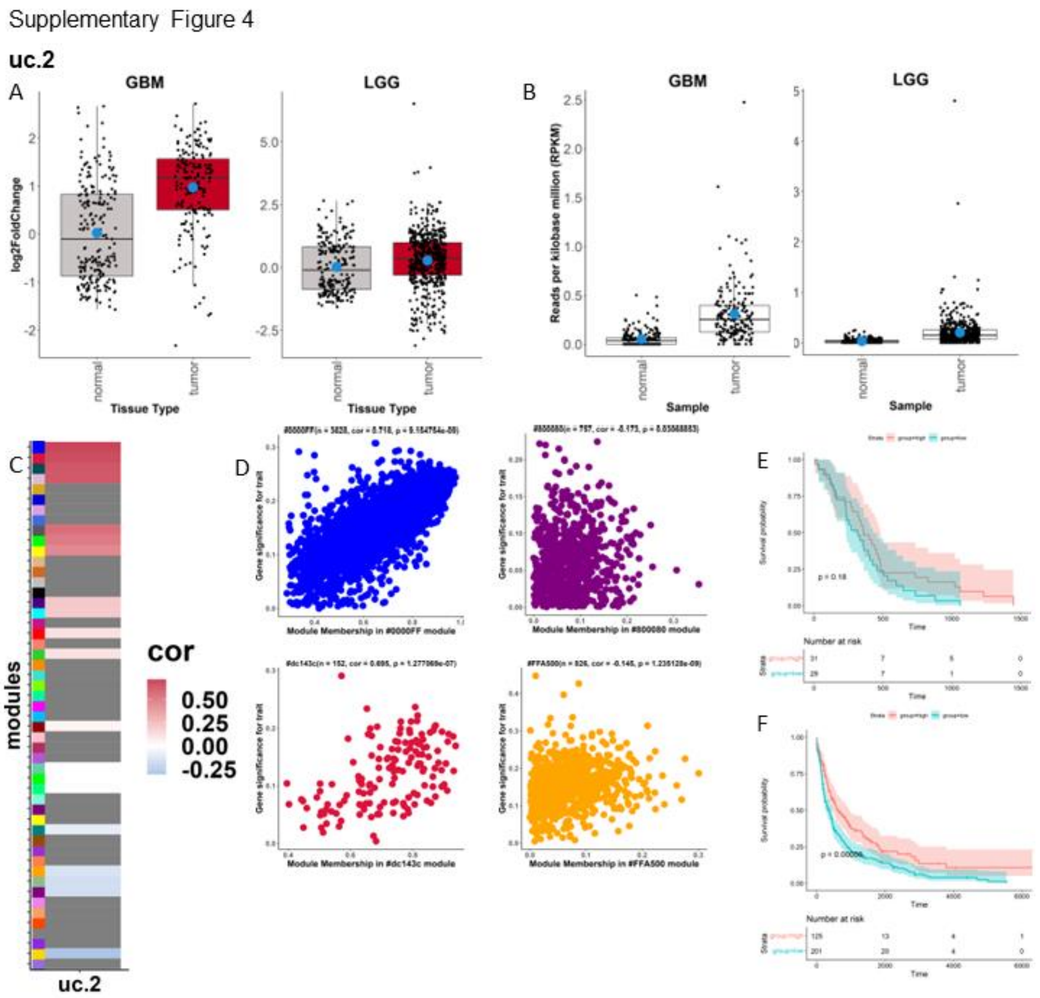

<u>Supplementary Figure 4. An exploration of a putative oncogenic TUCR, uc.2 in gliomas.</u> *A) Box- and dotplot showing uc.2 deregulation in GBM and LGG. Facets represent disease type. Red boxes represent upregulated TUCRs. Green boxes represent downregulated TUCRs. Gray boxes represent TUCRs that are not deregulated. B) Box- and dotplot showing uc.2 absolute expression in GBM and LGG. Facets represent disease type. C) Heatmap depicting uc.2 gene module association. Positive correlations are red, while negative correlations are blue, with weak correlations in white. Modules with no linkage are gray. D) Scatter plots depicting uc.2 association with top 3 positive (top row) and negative (bottom row) correlation modules. E) Kaplan-Meier showing uc.2 association with GBM prognosis. Red line represents the TUCR high expression group. Teal line represents the TUCR low expression group. F) Kaplan-Meier showing uc.2 association with LGG prognosis. Red line represents the TUCR high expression group. Teal line represents the TUCR low expression group. (Similar analyses and figures for all 481 other TUCRs available at www.abounaderlab.org/tucr-database/)*

bioRxiv preprint doi: https://doi.org/10.1101/2023.09.12.557444; this version posted March 20, 2024. The copyright holder for this preprint (which was not certified by peer review) is the author/funder. All rights reserved. No reuse allowed without permission.

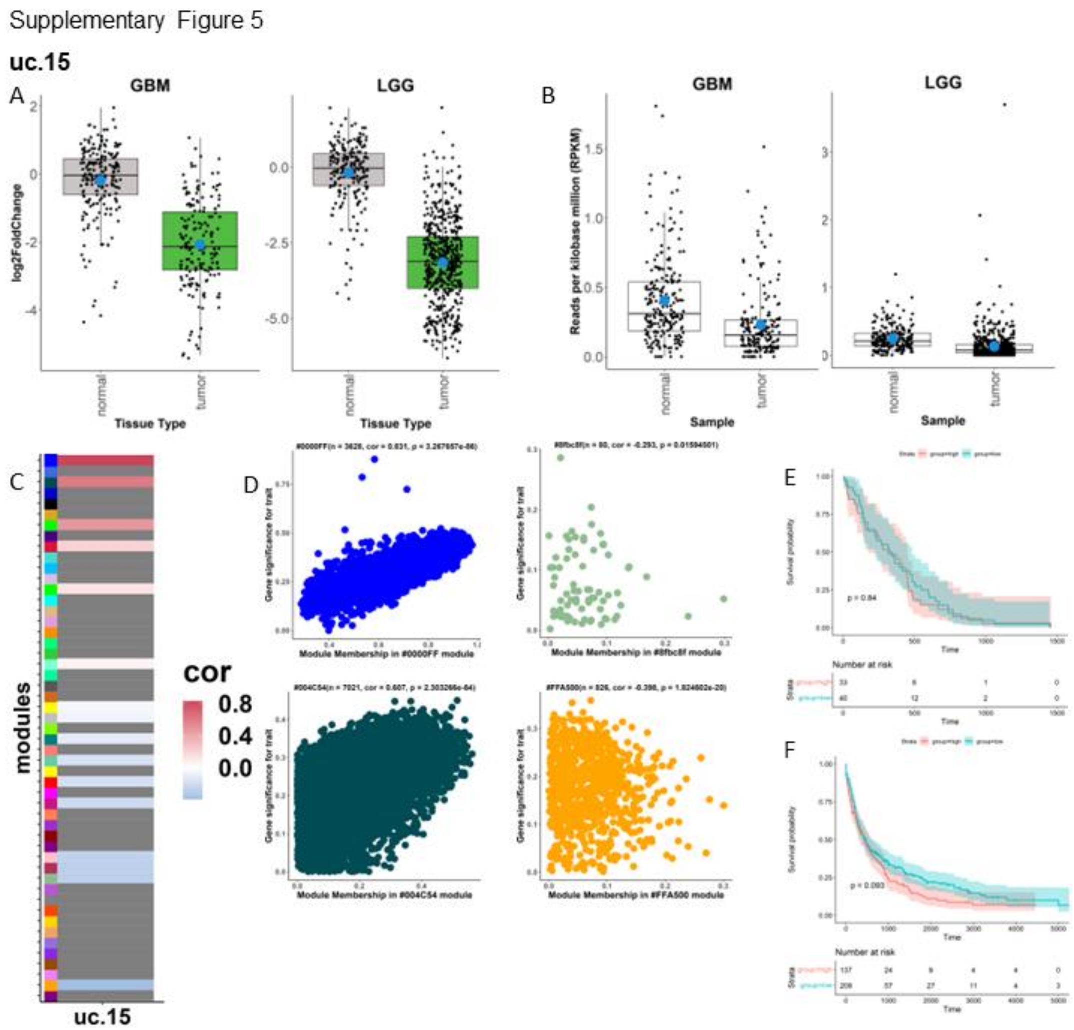

Supplementary Figure 5. An exploration of a putative oncogenic TUCR, uc.15 in gliomas. A) Box- and dotplot showing uc.15 deregulation in GBM and LGG. Facets represent disease type. Red boxes represent upregulated TUCRs. Green boxes represent downregulated TUCRs. Gray boxes represent TUCRs that are not deregulated. B) Box- and dotplot showing uc.15 absolute expression in GBM and LGG. Facets represent disease type. C) Heatmap depicting uc.15 gene module association. Positive correlations are red, while negative correlations are blue, with weak correlations in white. Modules with no linkage are gray. D) Scatter plots depicting uc.15 association with top 3 positive (top row) and negative (bottom row) correlation modules. E) Kaplan-Meier showing uc.15 association with GBM prognosis. Red line represents the TUCR high expression group. Teal line represents the TUCR low expression group. F) Kaplan-Meier showing uc.15 association with LGG prognosis. Red line represents the TUCR high expression group. Teal line represents the TUCR low expression group. (Similar analyses and figures for all other 481 TUCRs available at www.abounaderlab.org/tucr-database/)

bioRxiv preprint doi: https://doi.org/10.1101/2023.09.12.557444; this version posted March 20, 2024. The copyright holder for this preprint (which was not certified by peer review) is the author/funder. All rights reserved. No reuse allowed without permission.

Supplementary Figure 6

A

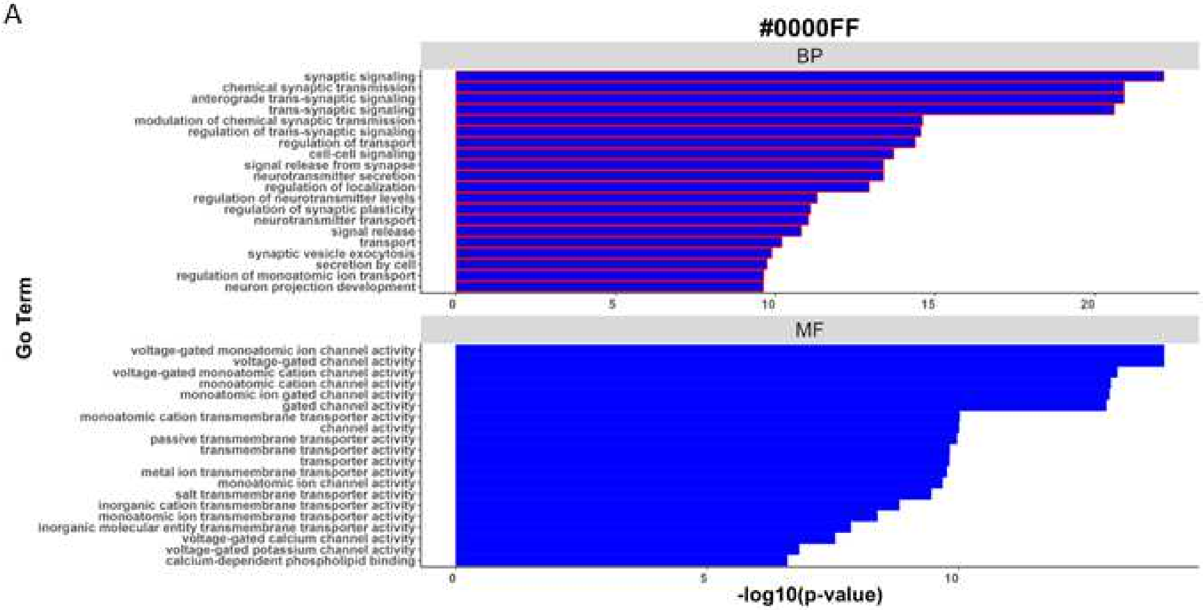

B

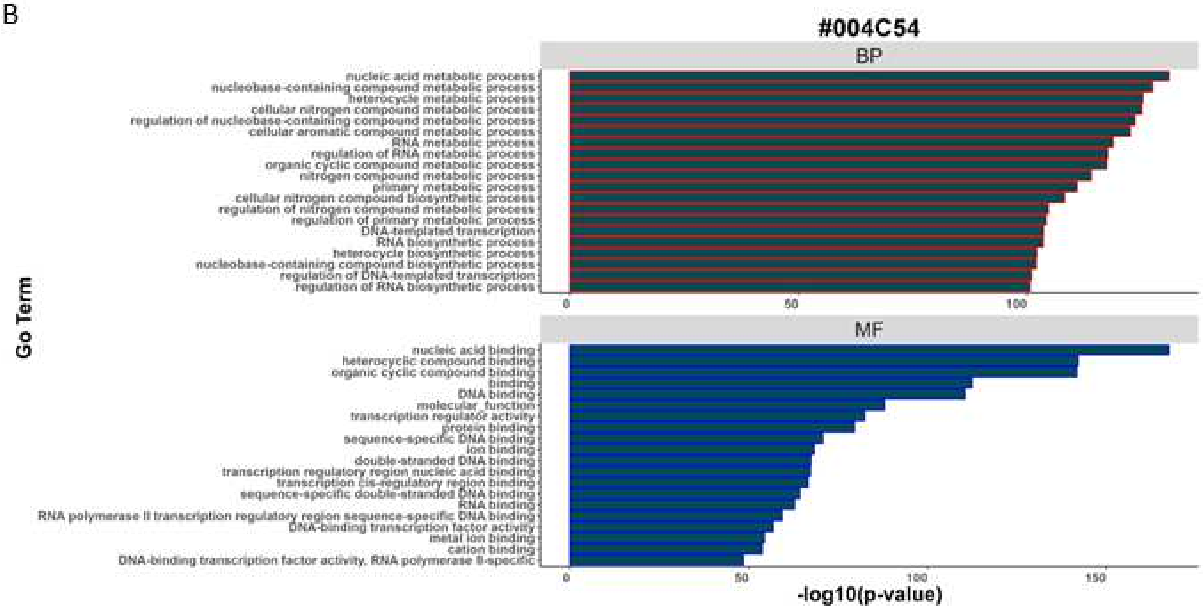

<u>Supplementary Figure 6. Top positively correlated TUCR modules in gliomas.</u> A) The #0000FF (blue) module is the most positively correlated with TUCRs. B) The #004C54 module (midnight green) is the second most positively correlated module with TUCRs.

bioRxiv preprint doi: https://doi.org/10.1101/2023.09.12.557444; this version posted March 20, 2024. The copyright holder for this preprint (which was not certified by peer review) is the author/funder. All rights reserved. No reuse allowed without permission.

Supplementary Figure 7

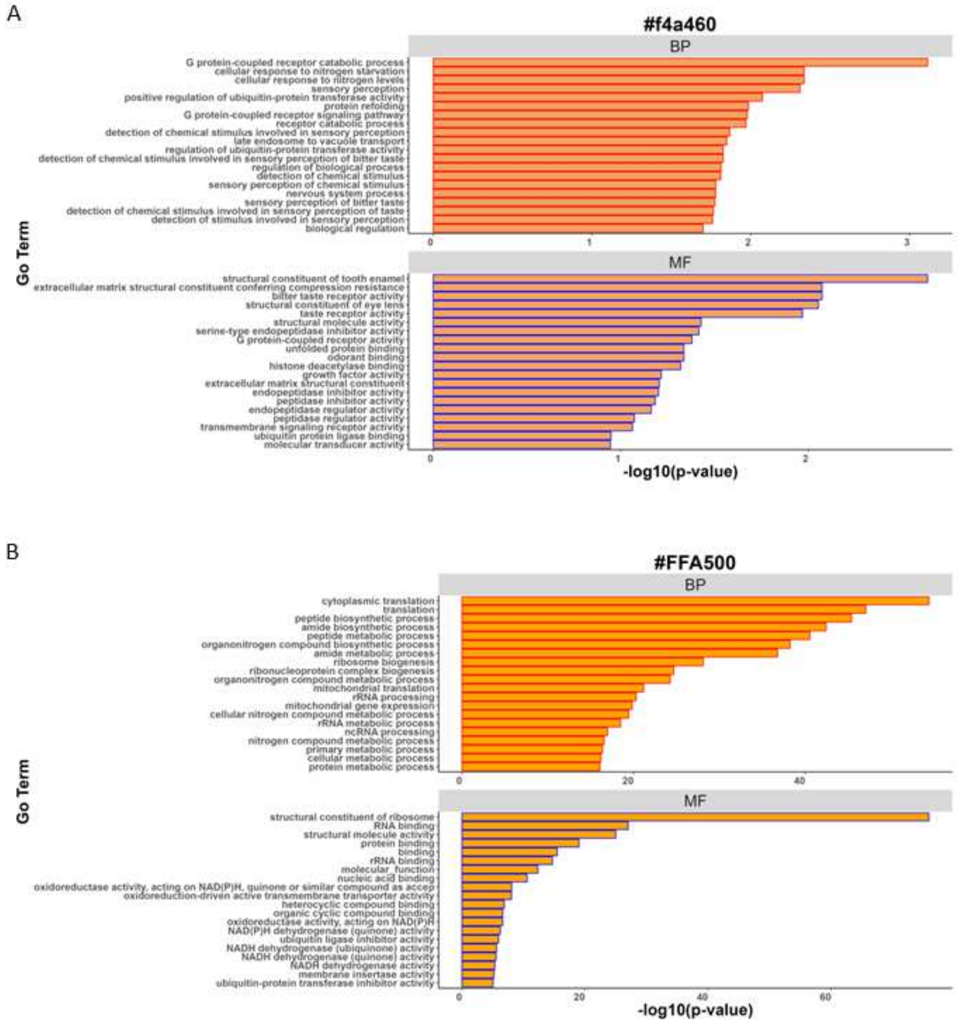

<u>Supplementary Figure 7. Top positively correlated TUCR modules in gliomas.</u> A) The #f4a460 (sandybrown) module is the most positively correlated with TUCRs. B) The #FFA500 module (orange) is the second most positively correlated module with TUCRs.

Supplementary Figure 8

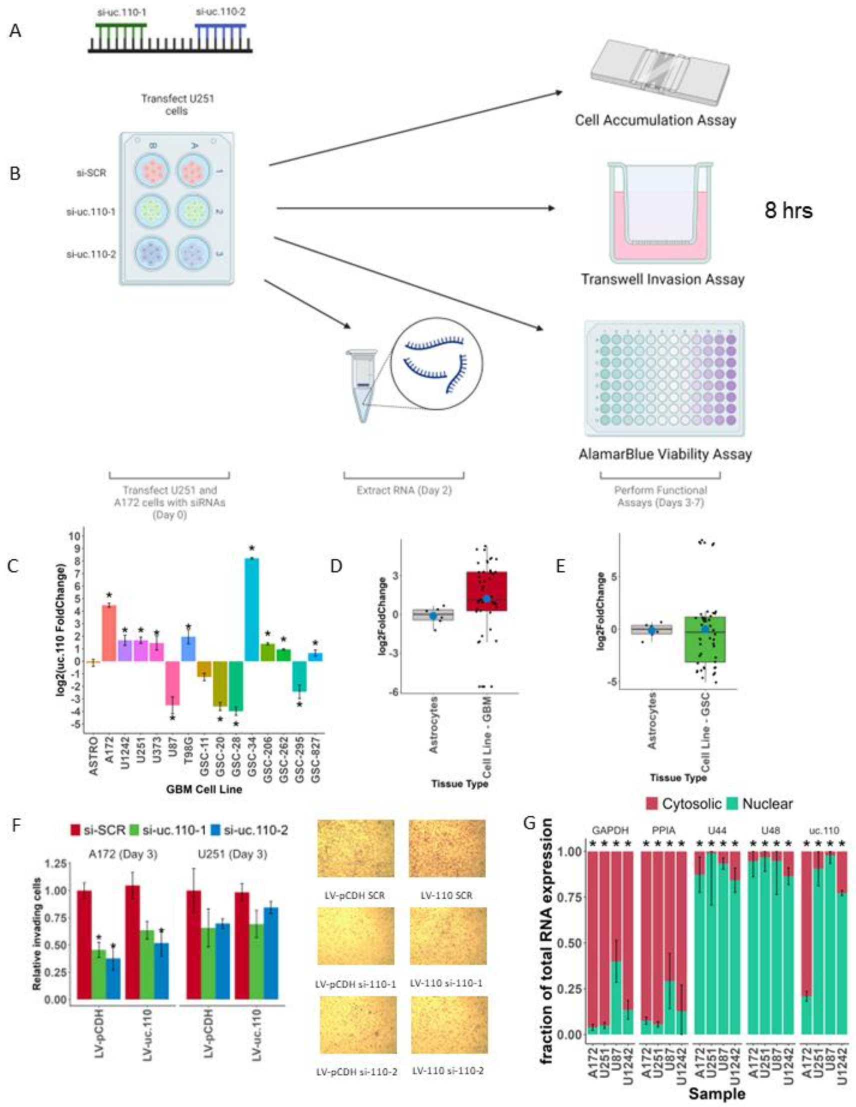

Supplementary Figure 8. The uc.110 TUCR operates as an oncogene (cont.) A) Cartoon depicting two siRNAs that target different regions of the uc.110 TUCR. One starts at nt 96/243 (blue), and the other at nt 195/243 (green). B) Cartoon schematic depicting transfection protocol. Cells were transfected with siRNAs using Lipofectamine 2000 at D0. RNA was collected at D2, and functional assays were performed from D3-D7. C) Bar graph depicting uc.110 upregulation in banked GBM cell lines. D) Boxplot representing uc.110 expression in pooled glioma adherent cell lines versus normal human astrocytes. Red boxes indicate an upregulated TUCR. D) Boxplot representing uc.110 expression in pooled glioma adherent cell lines versus normal human astrocytes. Red boxes indicate an upregulated TUCR. E) Boxplot representing uc.110 expression in pooled glioma stem cell lines versus normal human astrocytes. Green boxes indicate a downregulated TUCR. F) Bar graph depicting that the cell invasion phenotype is rescued in A172 and U251 cells with uc.110 overexpression in the presence of siRNAs. Images are representative of the listed sample. si-SCR = scrambled control siRNA (red), si-uc.110-1 = siRNA targeting uc.110 at nucleotide 96/243 (green), si-uc.110-2 = siRNA targeting uc.110 at nucleotide 195/243 (blue). G) Cell fractionation bar graph depicting that uc.110 is a predominantly nuclear RNA molecule, with cytosolic expression in A172s cells. Facets represent cytosolic (red) control genes (GAPDH, PPIA), nuclear (teal) control genes (U44, U48), and the uc.110 TUCR. * = $p < 0.05$

bioRxiv preprint doi: https://doi.org/10.1101/2023.09.12.557444; this version posted March 20, 2024. The copyright holder for this preprint (which was not certified by peer review) is the author/funder. All rights reserved. No reuse allowed without permission.

Supplementary Figure 9

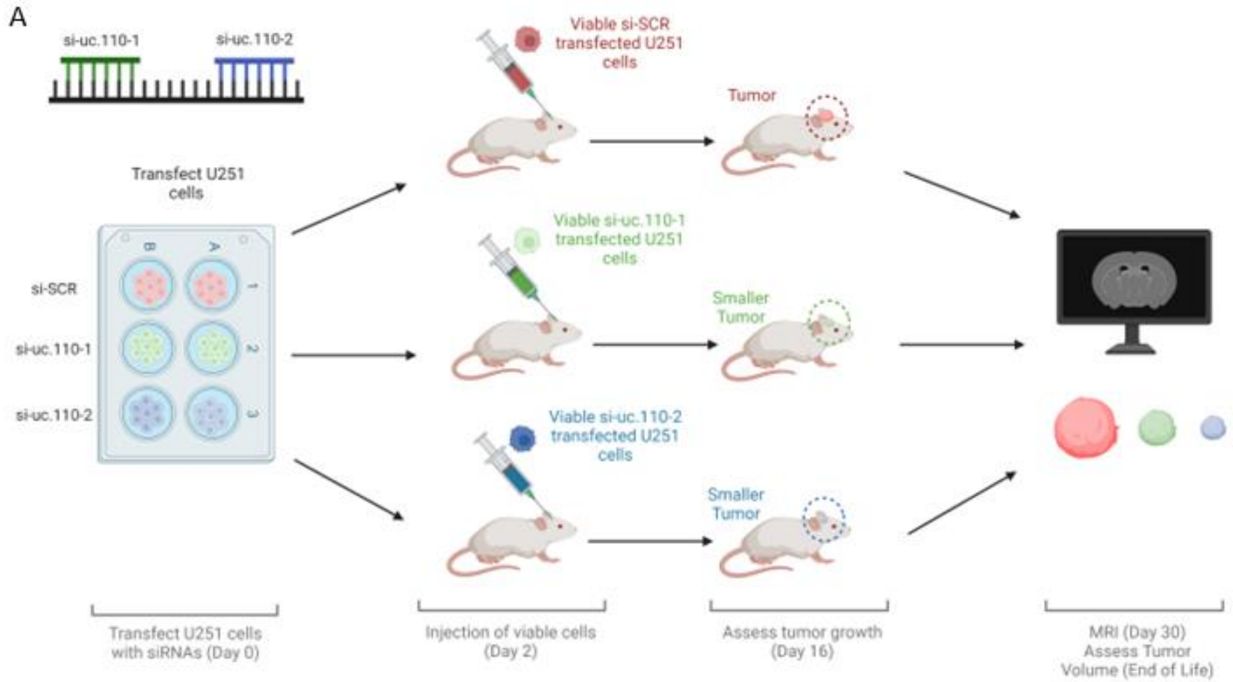

<u>Supplementary Figure 9. The uc.110 TUCR promotes tumor growth in vivo</u> A) Cartoon depiction of mouse experiment workflow. Cells were transfected with siRNAs using Lipofectamine 2000 at D0 and injected into mice at D2. Tumor growth was assessed weekly, starting at D16, via MRI.

bioRxiv preprint doi: https://doi.org/10.1101/2023.09.12.557444; this version posted March 20, 2024. The copyright holder for this preprint (which was not certified by peer review) is the author/funder. All rights reserved. No reuse allowed without permission.

Supplementary Figure 10

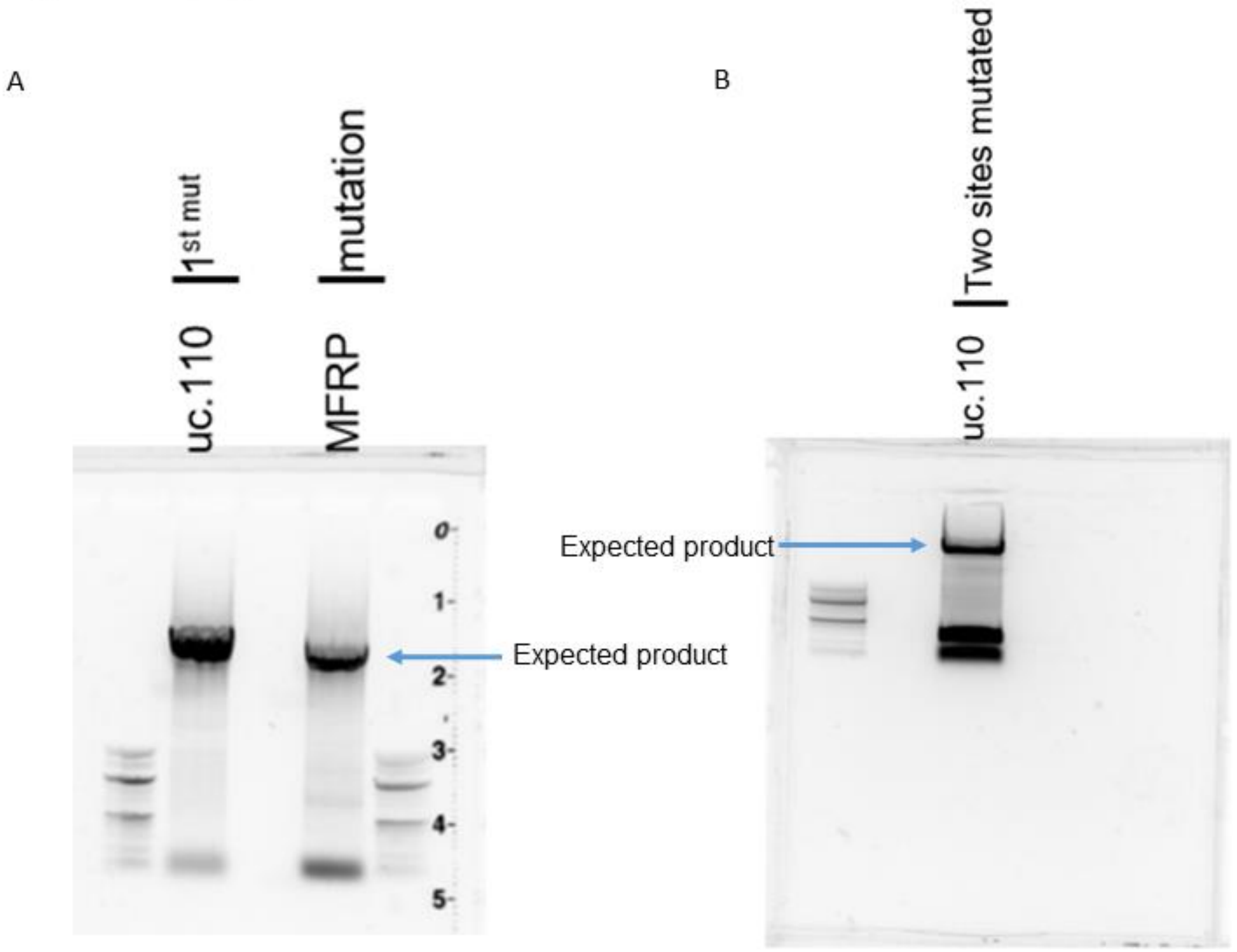

Supplementary Figure 10. PCR confirmation of mutation of miR-544 binding sites for MFRP and uc.110. *A) PCR gel showing expected products from uc.110 (first site) and MFRP mutations. B) PCR gel showing expected product from the second miR-544 binding site in uc.110.*

bioRxiv preprint doi: https://doi.org/10.1101/2023.09.12.557444; this version posted March 20, 2024. The copyright holder for this preprint (which was not certified by peer review) is the author/funder. All rights reserved. No reuse allowed without permission.

Supplementary Figure 11

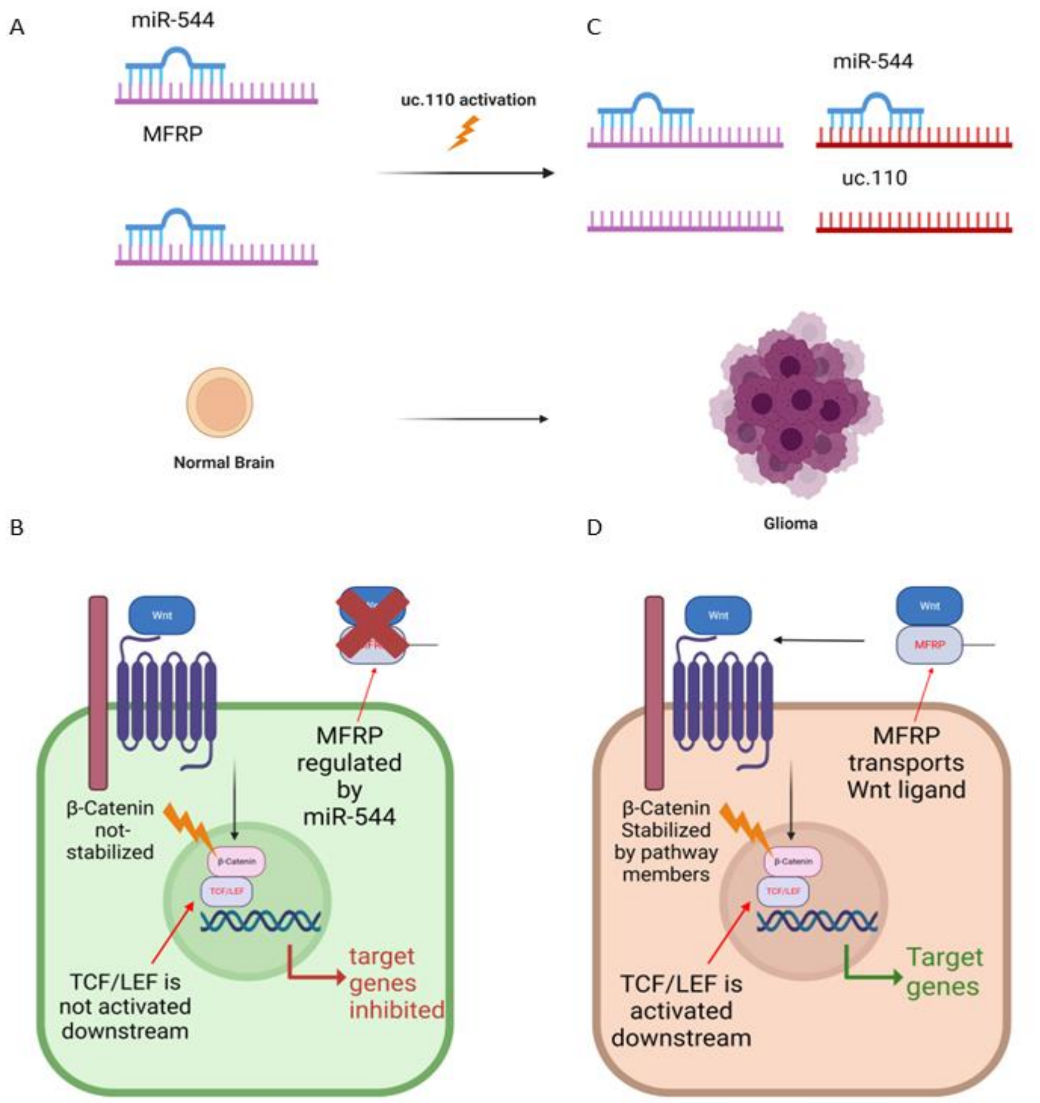

<u>Supplementary Figure 11. The uc.110 TUCR activates Wnt-signaling by sponging miR-544 from membrane frizzled related protein (MFRP) 3'UTR.</u> A) Schematic depicting model for miR-544 sponging by uc.110. B) Schematic depicting simplified repressed Wnt-signaling pathway. In the normal brain, MFRP is downregulated by miR-544 as depicted in 6A. C) Activation of uc.110 in glioma tumors leads to decreased bioavailability of miR-544. This increases the bioavailability of MFRP. D) Schematic depicting simplified activated Wnt-signaling pathway. When MFRP bioavailability is increased by uc.110 activation, as depicted in 6C, Wnt-signaling is also increased.
